# The Gut Microbiota Metabolite Urolithin B Improves Cognitive Deficits by Inhibiting Cyt C-Mediated Apoptosis and Promoting the Survival of Neurons Through the PI3K Pathway in Aging Mice

**DOI:** 10.3389/fphar.2021.768097

**Published:** 2021-11-15

**Authors:** Peng Chen, Fuchao Chen, Jiexin Lei, Gaohua Wang, Benhong Zhou

**Affiliations:** ^1^ Key Laboratory of Combinatorial Biosynthesis and Drug Discovery, Ministry of Education, Wuhan University School of Pharmaceutical Sciences, Wuhan, China; ^2^ Sinopharm Dongfeng General Hospital, Hubei University of Medicine, Shiyan, China; ^3^ Hubei Key Laboratory of Wudang Local Chinese Medicine Research, Shiyan, China; ^4^ Department of Endocrinology, Renmin Hospital of Wuhan University, Wuhan, China; ^5^ Department of Psychiatry, Renmin Hospital of Wuhan University, Wuhan, China; ^6^ Department of Pharmacy, Renmin Hospital of Wuhan University, Wuhan, China

**Keywords:** UB, d-Gal, aging, learning and memory, apoptosis, PI3K pathway

## Abstract

**Background:** Despite considerable advances in pharmacotherapy, more effective therapeutic interventions for aging-related neurodegenerative disorders (NDs), such as Alzheimer’s disease (AD), remain limited. Urolithin B (UB), one of the major subcategories of urolithins (microbiota metabolites) found in various tissues after ellagitannin consumption, has been shown to possess antioxidant, anti-inflammatory, and antiapoptotic effects. However, the neuroprotective effect of UB on brain aging in mice and its potential mechanisms were still unknown.

**Methods:** In the current research, we first assessed the ameliorative effects of UB on oxidative injury and apoptosis induced by H_2_O_2_ in neuro-2a cells. Then a subcutaneous injection of D-galactose in mice for 8 weeks was used to establish the aging model to evaluate the protective effects of UB. The capacity of memory and learning, alterations of hippocampus histology and corresponding molecular mechanisms were all evaluated.

**Results:** The D-gal-induced accelerated aging model *in vivo* demonstrated that UB could significantly ameliorate deficits in learning and memory by inhibiting the accumulation of advanced glycation end products (AGEs) and elevating the expression and activity of Cu, Zn-SOD and CAT. Furthermore, UB downregulated the c-Jun N-terminal kinase (JNK) signaling pathway and prevented cytochrome c release from isolated mitochondria, thereby inhibiting neuronal apoptosis during the aging process. More importantly, UB stimulation of aging mice activated ERK and phosphoinositide 3-kinase (PI3K), leading to neuronal survival along with Akt and p44/42 mitogen-activated protein kinase (MAPK) phosphorylation and activation.

**Conclusion:** In summary, UB effectively alleviated cognitive deficits and ameliorated brain aging-related conditions and could be considered a healthcare product to prevent aging-associated NDs such as AD.

## Introduction

Alzheimer’s disease (AD) is one of the most common progressive neurodegeneration diseases. It is characterized by the development of aging-associated dementia (progressive memory loss and cognitive dysfunction) and shows an increasing prevalence due to the progressive aging of the population (Schöll et al., 2016; Kellar and Craft, 2020). Despite the confirmation of pathological features such as extracellular β-amyloid (Aβ) plaques, excessive tau phosphorylation, and abnormal neurofibrillary tangles (NFTs) from the biochemical anatomical results of brain tissue in AD patients, the detailed mechanism of AD is still not completely understood, and effective disease-modifying treatments are largely limited and lacking (Qiang et al., 2017; Fortea et al., 2020). Recently, abundant literature has indicated that oxidative stress (OS) plays a key role in brain degeneration and the pathogenesis of AD (Ikram et al., 2021). The imbalance between excessive reactive oxygen species (ROS) generation and elimination can lead to impairments in various physiological and pathological processes, leading to a tremendous acceleration of the senescence process and the risk of AD occurrence and development. Thus, the focus of traditional AD pathomechanism research has gradually shifted toward an investigation of neuronal antioxidant defense.

D-galactose (D-gal) is a chemical compound (a reducing sugar) and a naturally occurring substance found in the human body. When it reaches a high concentration *in vivo,* D-gal is able to transform into aldose and hydroperoxide under the action of galactose oxidase, causing oxygen-derived free radical generation, which can also react with various reactive groups of amino acids and peptides to produce advanced glycation end products (AGEs) (Li et al., 2017). AGEs are the major resources of ROS and can further promote free radical generation, thereby impacting the function of biological systems (Chen et al., 2019). Evidence for the presence of a large body of aging models has verified that continuous chronic administration of a low dose of D-gal can produce aging-associated AD-like performance, including a disruption of deficits in learning and spatial memory, synaptic damage, neuronal loss, reduced activities of antioxidant enzymes and increased production of ROS (Lu et al., 2010; Wu et al., 2020a). Similarly, recent studies have shown that neuronal cell death is caused by a strengthened OS and could exacerbate motor and cognitive behavioral deficits (Bracko et al., 2020). Thus, the administration of D-gal to mice has been shown to be a more affordable method (few side effects and a high survival rate) to establish an accelerated aging animal model for various cognitive and behavioral function studies, as well as for drug development and drug efficacy testing.

To date, active natural metabolic products have been extensively tested as significant remedies and sources in anti-aging research and drug discovery in postponing age-related disorders such as AD and diabetes (Mozaffarian and Wu, 2018). Urolithin B (UB, Figure 2A, C_13_H_8_O_3_, 3-hydroxy-6H-benzo [c]chromen-6-one), also named 3-hydroxy-urolithin, is a type of metabolite that is produced by the human colon microflora from ellagitannins (ETs) and ellagic acid (EA) (Zheng et al., 2020). An update of *in vitro* and *in vivo* research has indicated that UB possesses a wide variety of pharmacological activities, such as antioxidative, anti-inflammatory, and antiapoptotic effects (Gao et al., 2020). Recently, the nervous system preventative function of UB has attracted the focus of many researchers, and it has been confirmed that UB can exert effective neurological modulating activity in various neuropsychological diseases, such as depression and schizophrenia (Yuan et al., 2015; Savi et al., 2018). These beneficial effects of UB may be due to its diverse pharmacological properties, such as its antioxidative, anti-inflammatory, and antiapoptotic potential (Gaya et al., 2018). However, little is known about the protective effect of UB in D-gal-induced memory impairment and degenerative brain disease.

Thus, the current study aimed to estimate the effect of UB on aging-associated cognitive deficiency and brain injury and to explore the underlying mechanisms by which UB inhibits cytochrome C-mediated apoptosis and promotes the survival of neurons via the PI3K pathway in the D-gal-induced aging process.

## Materials and Methods

### Materials

D-gal (Lot No. 1906754) was obtained from Sigma Aldrich Co. Ltd. (Germany, purity > 99%), and standard UB was purchased from Keygen Biotechnology Development Co., Ltd. (Nanjing, China, Lot No. 102346-214, purity > 99%). Commercial ELISA kits for monoamine oxidase (MAO, No. 20200506), acetylcholinesterase (AChE, No. 20200721), malondialdehyde (MDA, No. 20200623), superoxide dismutase (SOD, No. 20201216), total antioxidant capacity (T-AOC, No. 20200413), catalase (CAT, No. 20200326) and glutathione peroxidase (GSH-Px, No. 20200821) were purchased from Shanghai Yisheng Biotechnology Co., Ltd. (Shanghai, China). ELISA lab kits for interleukin-6 (IL-6, No. 20200625), tumor necrosis factor-α (TNF-α, No. 20200207) and interleukin-1 beta (IL-1β, No. 20200418) were purchased from Bioworld Technology Co., Ltd. (Nanjing, China). Bax, Bcl-2, cleaved caspase 3, Cu, Zn-SOD, CAT, p-p38, p38, p-JNK, JNK, Cyt C, Cox IV, GFAP, IBA1, PSD95, Syn, p-p44/42 MAPK (Thr202/Tyr204), p-p44/42 MAPK, p-AKT (Thr308), p-AKT (Ser473), AKT, Bad, P-Bad (Ser112) and P-Bad (Ser136) antibodies were all from Abcam (Shanghai, China).

### Determination of Antioxidant Capacity *in vitro*

#### DPPH Free Radical Scavenging Assay

DPPH was used to study the radical scavenging performance and antioxidant activity of medicinal plant natural active compounds (Ichikawa et al., 2019). Briefly, UB was prepared in 50% ethanol and diluted into the samples at different concentrations (40 μg/ml to 220 μg/ml). Next, 2 ml of freshly prepared DPPH solution (0.24 mM) was introduced into a 96-well microplate, and 2 ml of sample solution was added. Then, the mixture was shaken for several minutes to achieve a complete interaction and stored at 4°C in the dark. After 30 min, the absorbance values were determined at 519 nm. Vitamin C (VC) was chosen as the positive control group in the current study. The assay was conducted in triplicate, and the scavenging activity of the free radical DPPH as a percent (%) was determined according to the following equation: scavenging rate (%) = (Acontrol-Asample)/Acontrol × 100%.

#### ABTS^+^ Radical Scavenging Assay

The antioxidant activities of UB were determined by the ABTS cation radical scavenging capacity (Yao et al., 2015). Briefly, ABTS^+^ radical solution was prepared in the appropriate buffer mixed with equal volumes of 7 mM ABTS^+^ and 4.9 K_2_S_2_O_8_ aqueous solution incubated in the dark at room temperature for 10–18 h. This solution was diluted with 80% ethanol or phosphate buffer (pH 7.2–7.4) until the absorbance was 0.695–0.705 at a wavelength of 734 nm. Solutions were prepared fresh immediately prior to each assay replication, 0.3 ml of UB was allowed to react with 1.2 ml of the ABTS solution, and the absorbance was measured at 734 nm after a 10-min incubation at room temperature. VC was used as a positive control and observed with ABTS^**+**^. The ability of ABTS to scavenge cation radicals was determined as follows: scavenging rate (%) = [(Acontrol-Asample)/Acontrol]×100%.

#### OH- Scavenging Activity Test

The OH- scavenging activity assay was determined as described previously (Simunkova et al., 2021). The reaction mixture contained 0.1 ml of FeSO_4_ solution (9 mM), 0.1 ml salicylic acid dissolved in ethyl alcohol, 0.1 ml of H_2_O_2_ solution (8.8 mM) and 100 μl of different concentrations of UB. The total mixture was reacted at 37°C in a water bath for 20 min. The absorbance was recorded at 593 nm, and VC reagent served as the positive control solution. The final results were expressed using the following equation: Scavenging rate (%) = [(Acontrol-Asample)/Acontrol]×100%.

#### O- Scavenging Test

Furthermore, the O^**−**^ scavenging analysis was performed based on a previous method (Chen et al., 2020). In brief, 3 ml Tris-HCl buffer solution (pH 8.2) was incubated in a test tube, followed by the addition of 1 ml of UB at different concentrations (20 μg/ml to 220 μg/ml), and then these mixtures were mixed with 0.3 ml of pyrogallol solution (8 mm) reagent under conditions of rapid agitation and allowed to completely react for 10 min. In this assay, the OD values of the reaction mixture at 325 nm were recorded every 30 s after the addition of all reagents. The results were determined as follows: Scavenging rate (%) = [(Acontrol-Asample)/Acontrol]×100%.

### Cytoprotective Properties of the UB *in vitro* Cell Model

#### Cell Culture and Treatment

The mouse neuroblastoma N2a cell line (Neuro-2a) was purchased from Wuhan Baiyi Biotechnology Co., Ltd. (Wuhan, China) and cultured in low glucose Dulbecco’s Modified Eagle’s Medium (DMEM, Sigma, St. Louis, MO) supplemented with 10% fetal bovine serum (FBS, Gibco, Thermo Fisher Scientific) and 1% penicillin-streptomycin and then incubated at 37°C in a humidified atmosphere of 95% air and 5% CO_2_. Cells were seeded into plates and then randomly divided into five different groups: 1) blank group: normal medium; 2) H_2_O_2_ treatment group (250 μM); 3) H_2_O_2_ + UB (60 μg/ml) group: 250 μM H_2_O_2_ and 60 μg/ml UB; 4) H_2_O_2_ + UB (40 μg/ml) group: 250 μM H_2_O_2_ and 40 μg/ml UB; and 5) H_2_O_2_ + UB (20 μg/ml) group: 250 μM H_2_O_2_ and 20 μg/ml UB. Excluding the blank group, prior to exposure to H_2_O_2_ for 2 h, Neuro-2a cells were pretreated with UB and then incubated for another 24 h. The H_2_O_2_ and UB concentrations were selected based on previously published literature. Total proteins were extracted using the kit from Pierce based on the manufacturer’s instructions. The total level of protein was estimated using a bicinchoninic acid (BCA) protein assay kit (Bio-Rad, Stockholm, Sweden) on a microplate reader.

The viability of cells was assessed with the CCK-8 assay. Forty-eight hours following insult, the neurons were plated onto glass coverslips and then transferred to a 96-well plate. CCK8 solution was added to each well and incubated under standard culture conditions (1–4 h at 37°C). Finally, the cells were dissolved in 150 μl DMSO (Invitrogen, United States) after the medium was removed, and the absorbance of the samples collected from each well was detected at 492 nm using a multiwell plate reader. After oxidative insult (24 h after H_2_O_2_), cell cytotoxicity was assessed by the quantification of lactate dehydrogenase (LDH, Roche, Switzerland) at a wavelength of 490 nm following the manufacturer’s instructions. The results are presented as the relative value of LDH production versus the total protein.

#### ROS and Apoptosis Detection in Neuro-2a Cells by Flow Cytometry

After incubating the Neuro-2a cells for 24 h, the medium with phosphate-buffered saline (PBS) was replaced with PBS supplemented with glucose and DCFH-DA at 37°C for 30 min. Then, the medium was removed, and the cells were washed with warm sterile PBS and treated with H_2_O_2_ and various doses of UB (60, 40, and 20 µg/ml). Finally, the medium was aspirated and displaced cells were washed with PBS for three times. Then, the cell in each well was collected and ROS quantification by CellROX was detected using a flow cytometry. To confirm the neuroprotective effects of UB on neuron-2a cells, the rate of cell apoptosis induced by H_2_O_2_ was analyzed quantitatively by flow cytometry with an Annexin V/Fluorescein isothiocyanate (FITC) apoptosis detection kit. In detail, neuro-2a cells were first stimulated with an increasing concentration of UB solution (60, 40, 20 µg/ml) for 24 h. After treatment, detached cells were collected by centrifugation and rinsed three times with PBS. Subsequently, the cells were resuspended in 500 µl binding buffer, followed by staining with 10 µl Annexin V-FITC and 5 µl propidium iodide (PI) according to the manufacturer’s protocol. The apoptosis rates of neuro-2a cells were assessed by flow cytometry (FCM).

### Experimental Animals and Grouping Design *in vivo*

#### Experimental Animals

Six-to eight-week-old adult male C57BL/6 mice, each weighing 18–22 g, were sourced from the Experimental Animal Center of the School of Medicine, Wuhan University (SCXK (E) 2013-0004). The mice were placed in plastic cages under standard laboratory animal housing conditions (temperature of 20–22°C, relative humidity of 55–60% and a 12-h light-dark cycle). All animals were given free access to drinking water and pelleted food during the study period. All animal experiments were approved by the Ethics Committee of Animal Experiment and Laboratory Animal Welfare of Wuhan University (Hubei, China).

### Experimental Group and Drug Treatment *in vivo*

After 1 week of acclimation, the mice were randomly allocated to five groups each containing 12 animals as follows: normal control (Ctrl), D-gal-induced aging model (D-gal, 150 mg/kg/d), D-gal + high-dose UB (150 mg/kg/d, H-UB), D-gal + medium-dose UB (M-UB, 100 mg/kg/d), and D-gal + low-dose UB (L-UB, 50 mg/kg/d). The aging model was established by 8 weeks of daily subcutaneous injection (SI) of D-gal (150 mg/kg/d, dissolved in 0.9% sodium chloride (normal saline; NS) solution); the control group was administered NS in the same way (Li et al., 2017; Chen et al., 2019). UB reagent (150, 100, 50 mg/kg/d) dissolved in 8% Tween 80 was administered by intragastric gavage (IG) for the same experimental period (8 weeks), and the animals in the aging model and control groups received equal volumes of 8% Tween 80 in the same way (Chen et al., 2018). Furthermore, after this period (8 weeks of daily SI D-gal), a variety of behavioral tests were performed. The animals had free access to artificial granule food and water during the experiment. The dose of UB in the current study was selected based on a preliminary experiment in our lab. All procedures were conducted in accordance with the ARRIVE guidelines for mice. The animal experimental design is illustrated in Figure 2B.

In addition, to further investigate the molecular mechanisms of UB in aging mice, 2-month-old normal young mice (*n* = 24) and 12-month-old naturally senile mice (*n* = 24) were used. In detail, all mice were divided into four groups: Group 1: Ctrl mice of 2-month-old (*n* = 12); Group 2: UB (150 mg/kg; oral administration for 2 months) intervention for 2-month-old mice (*n* = 12); Group 3: Ctrl mice of 12-month-old (*n* = 12); Group 4: UB (150 mg/kg; oral administration for 2 months) intervention for 12-month-old mice. After 2 months of UB treatment, the behavioral performance of the animals was tested. Then, all mice were euthanized, and brain tissues were collected for subsequent pathologic and protein expression analyses to further evaluate the effect of UB on PI3K/Akt pathway regulation.

### Behavioral Tests

#### Open Field Test

The OFT was conducted using standard protocols according to a previous procedure with minor modifications (Chen et al., 2021). The animals were placed into a corner of the area and observed for approximately 5 min. After each trial, to clean the apparatus (remove any animal odor), a solution of 20% ethanol was used. The behavioral parameters were recorded as follows: duration and distance traveled in the center zone.

#### Morris Water Maze Test

To examine the effect of UB on spatial learning and memory function in aging mice, MWM was performed as reported previously (He et al., 2020). The MWM program and equipment were purchased from Shanghai Biowill Co., Ltd. A plastic, circular pool (diameter: 120 cm, height: 35 cm) filled with water (23 ± 2°C) to a depth of 30 cm was divided into 4 quadrants designated as north, east, west, and south. An escape platform (4–5 cm in diameter and 14 cm in height) was located in the center of one of the four equal-area quadrants of the water maze and was submerged below the surface of the water (1–1.5 cm). In the training trial, each mouse was placed in the water (in a random quadrant) and allowed 60 s to search for the platform. If the animals were unable to reach the platform after 120 s of swimming, they were gently guided to the platform or placed on it within 15 s before the platform was removed from the pool. Training was halted after 5 days (three trials per day), as all groups reached an average latency to find the hidden platform. After the training, all the mice were tested in the spatial probe trial (SPT). For the SPT, the platform was removed, and the latency to first visit the target zone as well as the staying time of rats in the target quadrant within 60 s were recorded. A computerized video tracking system was used to collect the movement data (escape latency, swim path, distance, and speed) displayed by each mouse.

#### Spontaneous Alternation Test (Y-Maze)

Working memory and exploration behavior were assessed using Y-Maze tests based on previously described methods (Ghafouri et al., 2016). The Y-maze consisted of three identical arms (each arm was 35 cm × 5 cm x 10 cm), and these arms were separated by 120°. In the training period, the novel arm was blocked out, and the animals were then free to swing in the start arm and other arms of the maze for 5 min. Upon completion of 5 min of exploration, the mouse was returned to its cage for 1 h. In the test phase, all arms were opened, and the animals were housed again in the start arm and allowed to explore the maze for 3 min. After completion of each trial, the time cost, distance traveled and the number of times the mouse entered the novel arm were all recorded. During the experiment, the maze arms were wiped with 70% (v/v) ethanol to avoid potential odor cues.

#### Tissue Sampling

Upon completion of the animal experiments, the mice were deeply anesthetized with an i.p. injection of sodium pentobarbital (100 mg/kg) and then transcardially perfused with 100 ml of ice-cold NS and 4% paraformaldehyde (PFA) into the heart (Lu et al., 2010; Chen et al., 2019). Thereafter, the brains were removed quickly, and the brain tissues were collected. One portion of the brains was fixed with 4% PFA in PBS for 72 h at RT and immersed in a solution of 30% sucrose for 12–24 h. The other portion was homogenized and stored at −80°C for further experiments. In addition, the effect of UB on the brain index (BI, brain/body weight ratio) was also investigated.

#### Biochemical Analysis

The activities of the brain antioxidative enzymes SOD, CAT and GSH-Px, MDA content, and protein concentration in brain tissues were all evaluated using commercially available highly sensitive ELISA kits. The brain tissue contents of IL-1β, TNF-α and IL-6 were measured using a standard ELISA kit method. All biochemical analyses were performed in accordance with the manufacturer’s instructions. Similarly, the MAO and AChE concentrations in brain tissue samples were also analyzed with ELISA kits.

#### Hematoxylin and Nissl Staining

Hematoxylin and eosin (H&E) and Nissl staining for the detection of neuronal loss were conducted. We deparaffinized the sections (4 μm) in xylene (2 × 5 min), hydrated them in graded ethanol solution and distilled water, and then incubated them with hematoxylin and eosin and 1% toluidine blue working solution. After gentle but thorough rinsing with distilled water, the sections were dehydrated and mounted with Permount. A normal microscope at 200x magnification (Olympus) was used to perform quantitative analysis of the number of surviving intact neurons. Five sections from each mouse were analyzed.

#### Golgi Staining and Morphological Analyses

Golgi staining is commonly used for studying the morphology of spinal motoneurons, and the FD Rapid GolgiStain™ kit (FD NeuroTechnologies, Inc.) was applied in this study. In detail, brain sections were fixed and then washed with fresh PBS several times, followed by incubation with Golgi-Cox (Santa Cruz, CA, United States) stain for 30 days, 30% sucrose solution for 2–3 days and sectioning (100 μm slice thickness) with a vibratome (VT1000S; Leica Microsystems, Wetzlar, Germany). Finally, the sections were silver stained. All samples were analyzed and photographed with a Leica [R] DM500 photomicroscope.

#### TUNEL Staining

TUNEL assays of brain tissue sections were conducted using an *in situ* cell death detection kit (Beyotime Biotech, China) based on the manufacturer’s protocol (Lu et al., 2010). Briefly, 4-μm-thick slides were dewaxed and then incubated with 10 μg/μl proteinase K (Roche, Basel, Switzerland) for 10 min at 37°C. Then, PBS was used to wash the sections, and endogenous peroxidase activity was blocked by incubation with H_2_O_2_ (10%) at room temperature for 15–20 min. Subsequently, the ultrathin sections were incubated with the TUNEL reaction mixture in a humidified chamber for 60 min at 37°C. The stop reaction was conducted by rinsing with 10 ml PBS solution and then adding 50 µl Converter-POD (vial 3) to the sample to promote the tailing reaction. After approximately 45 min at 37°C in a humidified chamber, 2 x standard saline citrate (SSC) was applied to terminate the tailing reaction. Unincorporated fluorescein-dUTP was then removed by washing the slides 3 times for 5 min each in PBS. The number of positive nuclei was counted in ten random high-power microscopic fields (×100 objective) per coverslip using a fluorescence microscope (Olympus).

#### Immunohistochemistry

The brain sections were fixed and processed for immunostaining as previously described (Hou et al., 2020). Briefly, the sections (5 μm) were dewaxed in xylene (10 min, twice) and rehydrated through decreasing concentrations of ethanol. After heat-induced antigen retrieval, to block endogenous peroxidase activity, the sections were incubated with 0.3% H_2_O_2_ for at least 30 min at 37°C. Then, the sections were washed with PBS, permeabilized in 1% Triton X-100 for 45 min at room temperature and blocked with 1% BSA/10% normal goat serum for 1.5 h. Afterwards, they were incubated at 4°C overnight with a rabbit anti-Iba-1 primary antibody (1:1000), GFAP (1:500), NeuN (1:1000) at RT for 48 h. To remove the primary antibody, the sections were washed three times with 1x TBS for 45 min, incubated with horseradish peroxidase (HRP)-conjugated secondary antibodies and then reacted with the avidin-biotin-peroxidase complex DAB 3,3′-diaminobenzidine DAB. Finally, a light microscope was used to obtain the immunohistochemistry images.

#### Electrophysiological Recordings

The electrophysiological tests were performed after the behavioral experiments and they were performed on the same behavioral animals. Preparation of the electrophysiological Rig and brain hippocampus slices was performed according to a previously published protocol (Konno et al., 2019). Briefly, after the mice were deeply anesthetized, they were rapidly decapitated to the hippocampi dissected and immersed in ice-cold artificial cerebrospinal fluid (ACSF) of the following composition (in mM): 124 NaCl; 2.6 KCl; 1.25 NaH_2_PO_4_; 25 NaHCO_3_; MgSO_4_ 1; 1.5 CaCl_2_; 9.5 glucose. ACSF was continuously gassed with 95% O_2_ and 5% CO_2_, pH 7.4. Sections (400 μm thick) were prepared using a McIlwain tissue chopper, allowed to recover at room temperature (20–22°C) for more than 1 h in Krebs solution, and then, according to previous descriptions of the CA1 stratum radiatum, field excitatory postsynaptic potentials (fEPSPs) were recorded. Furthermore, long-term potentiation (LTP, 10 trains with 2-s intervals of 200 Hz) was adapted from Dka B et al. (Konno et al., 2019) and recorded at a temperature maintained at 30–32°C.

#### Real-Time-Polymerase Chain Reaction

Quantitative real-time PCR was used to determine the expression of advanced glycation end products (AGEs), carboxymethyl lysine (CML), and receptor for advanced glycation end products (RAGE). Total RNA was extracted and purified from homogenized mouse brain tissue (40–100 mg) using TRIzol reagent. The purity and integrity of the extracted RNA was detected by a NanoDrop 2000C spectrophotometer (Thermo Scientific, Massachusetts, United States). One microgram of total RNA from each sample was subjected to first-strand cDNA synthesis using an Omniscript Reverse Transcriptase kit (Qiagen) and oligo (dT) primers according to the manufacturer’s recommendations (Promega, United States). Quantitative PCR was amplified with standard Power SYBR Green master mix (Applied Biosystems, United States). PCR reactions were performed following these steps: 1) denaturation at 95°C for 5 min; 2) 35 cycles of denaturation (30 s at 95°C); 3) followed by 52–56°C for 30 s, 72°C for 30 s, and 72°C for 1 min. Relative levels of target gene mRNA expression were normalized to β-actin, and the relative level of mRNA was calculated with the ΔΔ comparative threshold (Ct) method. The primers and their sequences used in this study were described in Table 1.

**TABLE 1:** Real-time PCR primer sequences.

Gene	Primer Sequence	Product size (bp)
AGEs	F: 5′-TGC​TAT​CAC​CCA​GGA​GCT​GT-3′	167
	R: 5′-GGA​GAG​AGG​ACC​TTC​CAA​GC-3′	
CML	F: 5-TCC​TCC​TAC​TCA​GGA​CGC​AA-3′	235
	R: 5′- CAG​TTG​GGA​TCT​TCC​GCT​CA-3′	
CEL	F: 5′-AAA​TTC​AAC​CAG​AGG​GGG​T-3′	128
	R: 5′-GGG​GTG​GAT​CTC​AGA​AAC​CA-3′	
RAGE	F: 5′-GGA​AGA​GGG​GCA​GAC​AGA​AC-3′	149
	R: 5′-GAG​GAC​CTT​CCA​AGC​TTC​AGT-3′	
β-actin	F: 5′-CTC​AGA​GCA​AGA​GGC​ATC​C-3′	134
	R: 5′-GTG​CTC​CCA​GTT​GGT​GAG​AAT​G-3′	

#### Western Blotting

Whole protein samples obtained from brain tissues and cells were extracted using RIPA buffer extraction reagent containing PMSF (1:1000; Sigma, St. Louis) or cocktail. The protein concentration was determined by the BCA protein assay kit (Biosharp, China). An equal amount of protein sample was subjected to 10% sodium dodecyl sulfate polyacrylamide gel electrophoresis (SDS-PAGE) gels and subsequently electroblotted onto polyvinylidene fluoride (PVDF) membranes. Then, the membranes were blocked by incubation for 2 h at room temperature (RT) with 5% nonfat milk in Tris-buffered saline with Tween^®^ 20 (1x TBS-T). After washing the membranes three times (4 × 15 min) using 1x TBST, the membranes were probed with specific primary antibodies (Bax, Bcl-2, cleaved-caspase 3, Cu, Zn-SOD, CAT, p-p38, p38, p-JNK, JNK, Cyt C, Cox IV, GFAP, IBA1, PSD95, Syn, p-p44/42 MAPK (Thr202/Tyr204), p-p44/42 MAPK, p-AKT (Thr308), p-AKT (Ser473), AKT, Bad, P-Bad (Ser112) and P-Bad (Ser136) antibodies) and incubated at 4°C overnight in a humidified chamber. On the next day, the blots were subsequently incubated with HRP-conjugated secondary antibodies. The protein bands were then visualized using enhanced chemiluminescence (ECL) detection reagents (GE Healthcare, Little Chalfont, United Kingdom).

#### Data Processing and Statistical Analysis

All results are presented as the mean ± standard deviation (SD). The experimental results were evaluated a priori using the Shapiro-Wilk normality test for Gaussian distribution. All data were statistically analyzed with one-way analysis of variance (ANOVA), followed by Bonferroni’s post hoc test corrected for multiple comparisons. The results were considered statistically significant when *p*-values were less than 0.05. GraphPad Prism version 6.1 (GraphPad Software, California, United States) was used to perform the statistical analysis.

## Results

### Radical Scavenging Ability of UB *in vitro*

OS has long been implicated as a potential proximal physiological cost of reproduction during the lifespan of individuals (Ikram et al., 2021). It has been confirmed that OS is determined by a negative regulator of ROS and has long been thought to be one of the most significant elements to be considered in accelerating chronic disease progression, such as Alzheimer’s and Parkinson’s disease, diabetes, aging, cancer, muscular dystrophy and heart disease (Lu et al., 2010; Wu et al., 2020a). To evaluate the antioxidant capacity of UB, we measured the scavenging rates of four kinds of free radicals, DPPH, ABTS^+^, O_2_^−^ and OH^−^, which are commonly used to measure the total antioxidant capacity of chemical drugs and natural products. The experimental results showed that UB had a strong ability to scavenge ABST free radicals and that the radical-scavenging effect was dose-dependent. The 50% effective concentration (EC_50_) of UB was 316.18 ± 1.85 mmol/L, which was lower than that of VC (526.24 ± 3.18 mmol/L) (Supplementary Figure S1A). Furthermore, we found that the EC_50_ of UB against DPPH was 295.41 ± 2.36 mmol/L, whereas that of VC was 446.25 ± 1.78 mmol/L (Supplementary Figure S1B), indicating that UB also possessed strong activity for scavenging DPPH. We also measured the scavenging ability of UB on OH^−^ and O_2_^−^, as shown in Supplementary Figures S1C,D. The EC_50 values_ of UB and VC for OH^−^ (O_2_^−^) were 306.28 ± 4.61 (495.32 ± 3.28) mmol/L and 540.16 ± 2.52 (874.39 ± 1.48) mmol/L, respectively, suggesting that UB had good radical scavenging potential toward OH^−^ and O_2_^−^ and was comparable to VC.

### Protective Effect of UB on Neuron-2a Cell *in vitro Model*

#### UB Protects Against H_2_O_2_^−^ Induced Neuron-2a Cell Viability, Cytotoxicity, and Apoptosis

**FIGURE 1:**
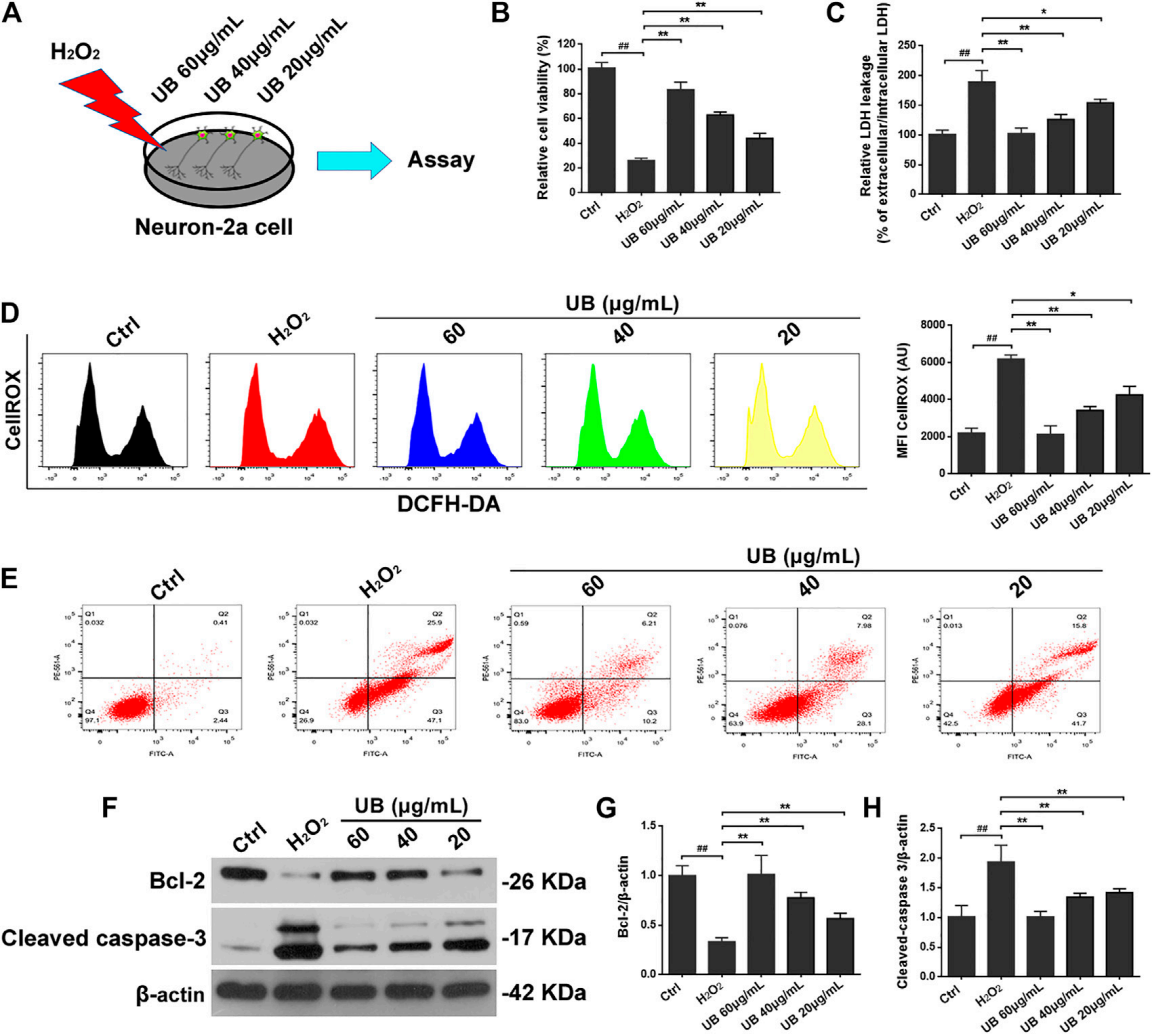
**(A)** The schematic diagram of the cell experimental design for H_2_O_2_-induced Inflammatory injury model and UB treatment. The protective effect of UB on neuron-2a cells against H_2_O_2_-induced viability **(B)** and cytotoxicity **(C)**. **(D)** UB inhibited ROS accumulation in neuron-2a cells, as determined by flow cytometry. **(E)** The anti-apoptotic effect of UB against H_2_O_2_-induced neurotoxicity in neuron-2a. **(F)** The expression of cleaved caspase-3 and Bcl-2 was detected by western blotting. Quantification analysis of cleaved caspase-3 **(G)** and Bcl-2 **(H)**. Data were normalized to β-actin protein expression. All results are described as means ± SD (*n* = 3 per group). ^#^ *p* < 0.05 and ^##^ *p* < 0.01 vs the control group; ^*^ *p* < 0.05 and ^**^ *p* < 0.01 vs the H_2_O_2_-treated group.

To measure the effects of UB on neuron-2a cell viability and cytotoxicity, CCK-8 and LDH assays were used in this study. As shown in Figure 1B, administration of H_2_O_2_ to control neuron-2a cells significantly reduced viability compared with that of the control group. However, this event was significantly reversed in the UB intervention groups compared with the H_2_O_2_-induced damaged model group under a significant dose-effect relationship (*p* < 0.05 or *p* < 0.01). Conversely, compared with the control group, there was a significant increase in LDH release after H_2_O_2_ treatment of neuron-2a cells. Interestingly, LDH activity in neuron-2a cells was significantly attenuated in the UB-treated group (Figure 1C).

Accumulating evidence has demonstrated that mitochondrial ROS are potential mediators of cell apoptosis, since H_2_O_2_ can cause apoptosis, which may be associated with the production of endogenous ROS (Chen et al., 2019; Zheng et al., 2020). Thus, we measured the H_2_O_2_-induced generation of ROS in neuron-2a cells using DCFH-DA, a common technique for directly detecting the redox state of cells. As presented in Figure 1D, compared with the control group, there was increased ROS production (approximately 108.9%) in the H_2_O_2_-treated group. However, after intervention with UB at 60 μg/ml, ROS production was dramatically decreased by 51.08%, while UB at 40 and 20 μg/ml decreased ROS production by 38.73% and 32.48, respectively, indicating a possible antioxidant effect of UB.

To further explore whether UB could inhibit neuron-2a cell apoptosis in the H_2_O_2_-induced neuronal *in vitro* model of oxidative injury, Annexin V-FITC staining was used. The results from the apoptosis experiments revealed distinct apoptosis (the apoptosis rate was approximately 73.45%) after neuron-2a cells were subjected to H_2_O_2_ exposure for 2 h (Figure 1E). However, the apoptosis rate was significantly reduced (*p* < 0.05 or *p* < 0.01) when UB-treated groups were compared with the H_2_O_2_-interposed model group, and the high-dose UB group (with a concentration of 60 μg/ml) possessed the best experimental results on apoptosis remission. These data showed that administration of UB could validly abolish H_2_O_2_-induced apoptosis in neuron-2a cells. Furthermore, we also inspected the expression of proteins associated with apoptosis, such as Bcl-2 and cleaved caspase-3, in a neuron-2a cell injury model induced by H_2_O_2_. As described in Figures 1F–H, increased protein levels of cleaved caspase-3 and decreased expression of Bcl-2 were observed in the H_2_O_2_-treated group compared with the vehicle control group. However, all of these indicators were effectively relieved after incubation for 24 h with the naturally occurring compound UB (*p* < 0.05 or *p* < 0.01).

### *In vivo* Results of the Ameliorative Effect of UB on D-Gal-Treated Aging Animals

#### Effects of UB on Changes in Body Weight and BI in Aging Mice

**FIGURE 2:**
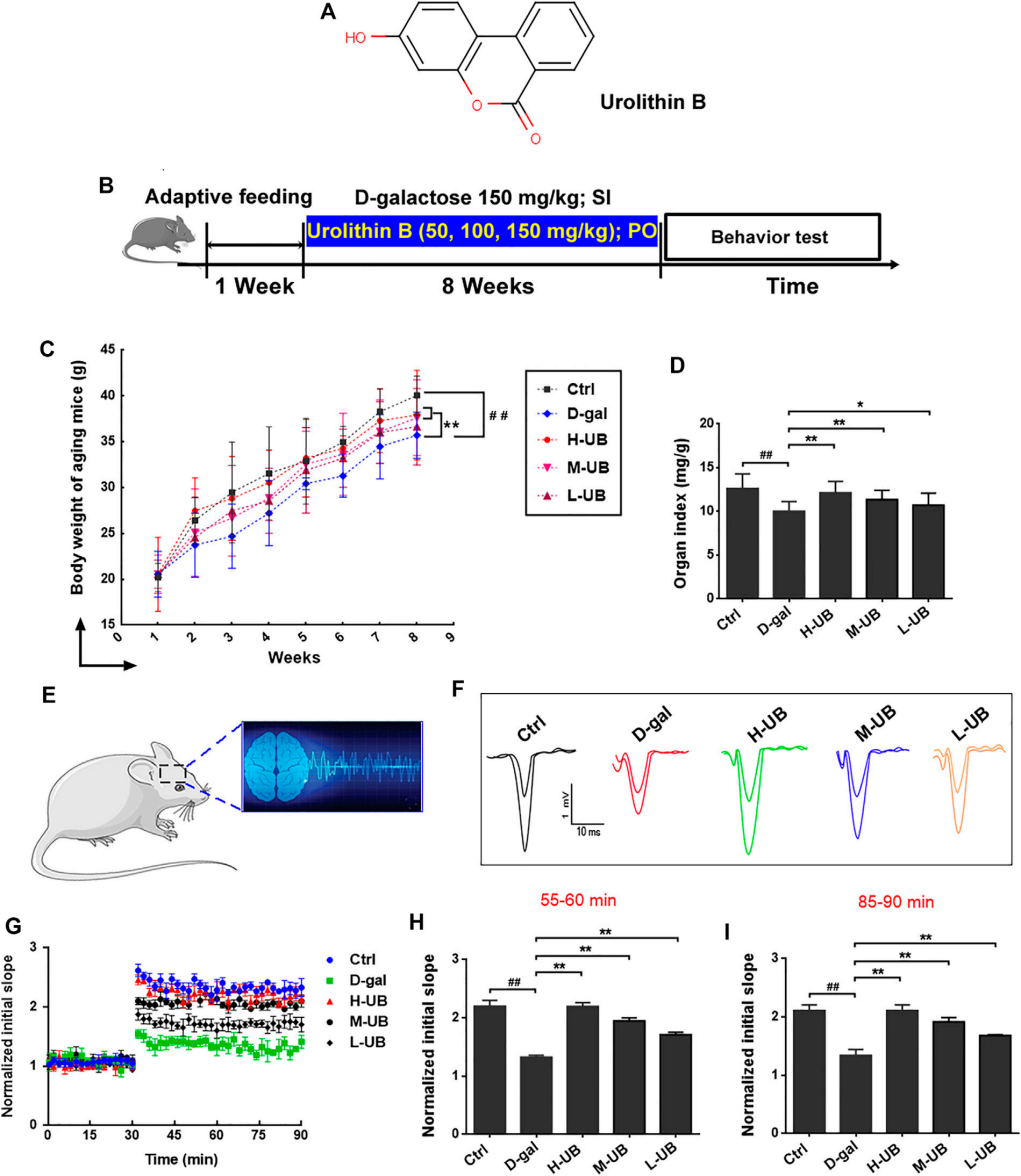
**(A)** Molecular structure of UB. **(B)** Schematic diagram of the experimental design for the D-gal-induced aging model and UB treatment. **(C)** Effect of UB on body weight (C) and brain index **(D)** in all groups (*n* = 12 per group). **(E)** A graphical drawing showing electrophysiological recordings. **(F)** Representative fEPSP traces. **(G)** The slope of the fEPSP was measured for 90 min. The data represent averaged increases in the slope of the fEPSP at t = 55–60 **(H)** and 85–90 **(I)** min relative to baseline (*n* = 6 per group). All results are described as means ± SD. ^#^ *p* < 0.05 and ^##^ *p* < 0.01 vs the control group; ^*^ *p* < 0.05 and ^**^ *p* < 0.01 vs the D-gal-induced aging group. SI: Subcutaneous Injection; PO: Per Oral.

The mice in the control group were active and rejuvenative, the hair of the mice was pitch-black and shiny, and no symptoms of depression or lethargy were found from week 5. In contrast, the animals in the aging model group were anorectic, dull, unresponsive, and exhibited hair loss when compared with the healthy controls (Chen et al., 2018). Nevertheless, these deteriorated symptoms alternated in mice of all doses of the UB groups. The dynamic changes in body weight were also measured during 8 weeks of feeding. As shown in Figures 2C,D, at the end of the animal experiment, the body weight and BI in D-gal-treated aging mice were significantly decreased compared with those of mice in the control group (all *p* < 0.01), but long-term intervention with UB for 8 weeks attenuated these decreases compared with those in the D-gal-induced aging model group.

#### Effects of UB on Improving Cognitive Deficits in Aging Mice

**FIGURE 3:**
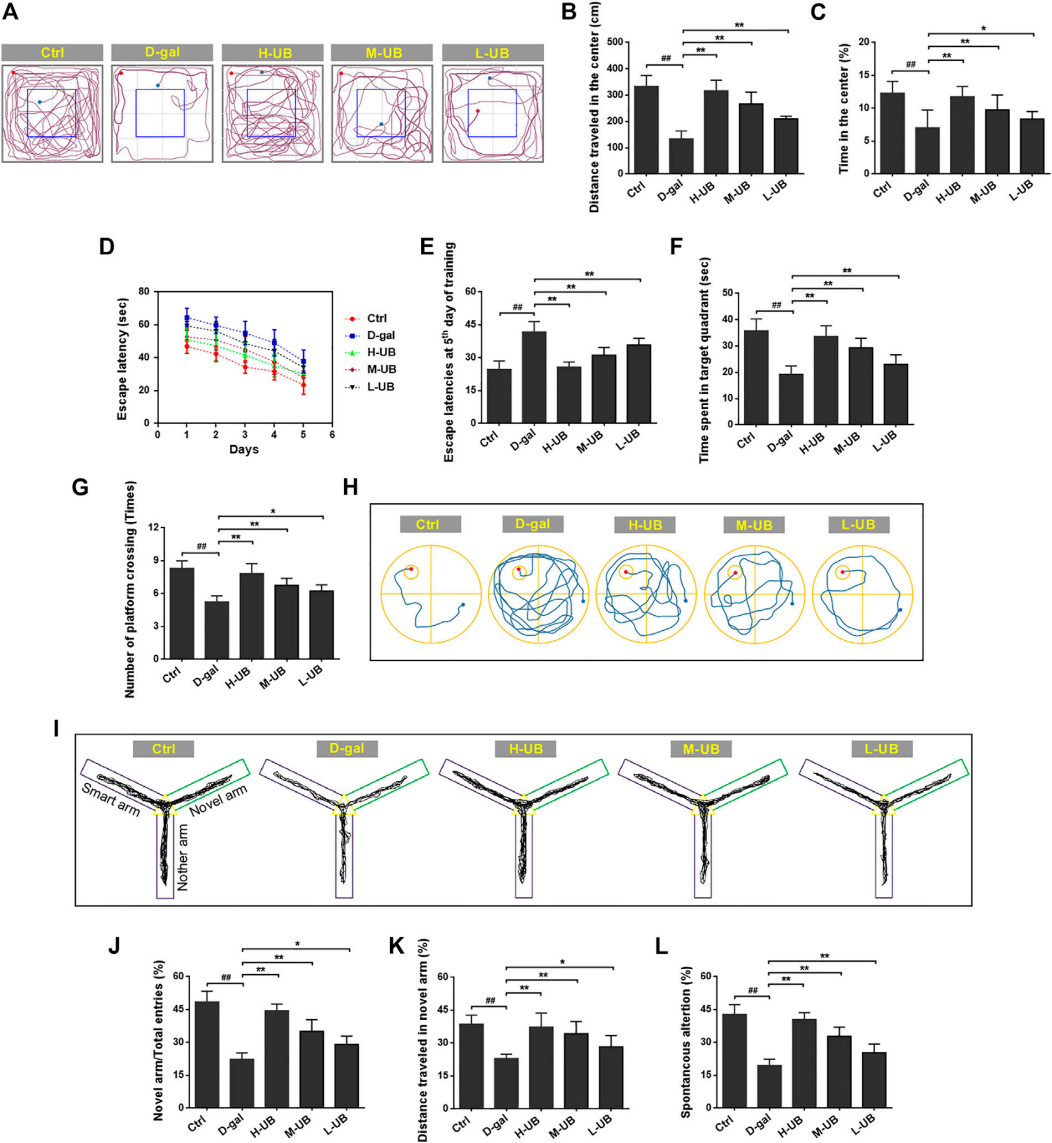
Effect of UB on memory impairment in D-gal-induced aging mice. **(A)** Representative trajectory representing the areas where the animals spent time in the OFT. **(B)** The total distance the animal traveled in the center of the arena. **(C)** The mean time the animals spent within the center of the arena. **(D)** Escape latencies of aging mice in training trials. **(E)** Escape latencies of aging mice on day 5 in the training trials. **(F)** Time spent in the target quadrant during the probe test. **(G)** The number of platform location crosses by mice. **(H)** Representative traces on the last day of the probe test. **(I)** Representative trajectory representing the areas where the animals spent time in the Y maze test. **(J)** The frequency of entries into the novel arm. **(K)** Total distance traveled in the novel arm. **(L)** Spontaneous alteration during the Y maze. All results are described as means ± SD (*n* = 12 per group). ^#^ *p* < 0.05 and ^##^ *p* < 0.01 vs the control group; ^*^ *p* < 0.05 and ^**^ *p* < 0.01 vs the D-gal-induced aging group.

To further evaluate the effect of UB on the spontaneous locomotor activity of animals, an open-field test was conducted. The experimental data showed a reduced distance traveled and less time spent in the central square in D-gal-induced aging mice compared with healthy controls (*p* < 0.05 or *p* < 0.01), indicating that D-gal administered to mice could successfully induce spontaneous activity defects (Figures 3A–C). More interestingly, all doses of UB treatment displayed a noticeably greater tendency to move further, and these mice spent more time in the central area, indicating that UB could ameliorate the spontaneous open-field activity imperfection.

It has been reported that D-gal can cause impaired spatial and sequential learning; thus, MWM was employed to measure the protective effect of UB on age-related mouse spatial learning and memory deficits. As shown in Figures 3D,E after 8 weeks of D-gal administration, the escape latency was significantly prolonged from the first day of training to the end of the training period [*F* (4, 55) = 15.51, *p* < 0.01], and this increase was prominently attenuated compared with that in the D-gal-treated aging model group. During the probe trial in this study, the aging mice spent less time in the goal quadrant, and the number of platform crossings was also significantly decreased in these mice compared with the healthy controls (both *p* < 0.01, Figures 3F–H). Nevertheless, treatment with UB significantly restored the above poor conditions. These results suggested an ameliorating effect of UB on D-gal-induced impaired learning ability and memory performance in the MWM test.

We also performed Y-maze behavioral tests and measured the levels of neurotransmitters in mice exposed to D-gal. The findings revealed that D-gal-treated aging mice spent significantly less time and traveled a shorter distance in the novel arm of the Y maze than the normal control mice (*p* < 0.05 or *p* < 0.01, Figures 3I,K). However, long-term UB administration significantly increased the time spent and distance traveled in the novel arm compared with aging mice. Similarly, a decrease in entry into the novel arm and spontaneous alterations were found in D-gal-treated aging compared with control mice (Figures 3J,L). These findings showed that D-gal might cause a deficiency in spatial memory in aging mice. Such a deficiency in memory was significantly inhibited in the UB-treated mice, indicating that it displayed good effects on the development of discriminative learning and working memory.

#### Effects of UB on Impairment of Hippocampal LTP in Aging Mice

To further verify the impaired learning and memory ability of aging mice, we used an *in vitro* electrophysiological technique to detect abnormalities in LTP in the hippocampus of animals. It has been confirmed that LTP is important for evaluating learning ability and memory in animals, in which the hippocampus receives high-frequency stimulation, leading to LTP of synaptic strength, which can last for hours or even days. In this study, after high-frequency stimulation, the slope of all groups increased more than 30%, indicating that the induction was successful (Figure 2G). Then, we calculated the slopes from 55 to 60 min and 85–90 min, and we found that the slope in the aging group decreased compared with that in the control group [55–60 min: *F* (4, 25) = 104.7, *p* < 0.01; 85–90 min: *F* (4, 25) = 47.31, *p* < 0.01; Figures 2H,I]. However, the slope was increased in UB-treated mice in comparison to the control group. These data indicated that UB could improve the induction of hippocampal LTP and improve learning and memory impairment in aging mice.

#### Effects of UB on the Biochemical Criteria of the Whole Brain of Aging Mice

The results of experiments performed in D-gal-induced aging mice are presented in Supplementary Figure S2. We observed significant production of AChE and MAO in aging model mice compared with healthy animals [AChE: *F* (4, 25) = 12.03, *p* < 0.01; MAO: *F* (4, 25) = 22.32, *p* < 0.01]. It is worth noting that UB supplementation in aging mice was associated with an increase in the levels of AChE and MAO compared with those in D-gal-treated aging mice (*p* < 0.05 or *p* < 0.01, Supplementary Figures S2A,B). These data indicated that UB could adjust the balance of the AChE and MAO levels, contributing to improving the behavioral impairment and cognitive disorder caused by D-gal.

We measured key antioxidants, including enzymatic and nonenzymatic antioxidants such as SOD, CAT, GSH-Px, and T-AOC, and oxidative stress-induced MDA levels. There was a significant decrease in GSH-Px levels and activities of SOD, CAT and T-AOC in the brains of model mice when compared with control mice (all *p* < 0.01), and UB could significantly rescue these biochemical indicators, which was significantly different from D-gal-alone-treated mice [SOD: *F* (4, 25) = 30.14, *p* < 0.01]; CAT: *F* (4, 25) = 40.02, *p* < 0.01; GSH-Px: *F* (4, 25) = 22.73, *p* < 0.01; T-AOC: *F* (4, 25) = 27.74, *p* < 0.01; Supplementary Figures S2C–F]. In addition, significantly increased MDA levels were observed in the model aging group compared with the control group, and the administration of UB caused a significant decline in MDA levels in the brain tissue of D-gal-treated mice [*F* (4, 25) = 48.56, *p* < 0.01; Supplementary Figure S2G]. In brief, these findings indicated that UB could exert good antioxidant function in D-gal-induced aging mice.

Subsequently, to verify whether the memory improvement effect of UB in aging mice was associated with alleviating brain inflammation, we observed proinflammatory cytokines in the brain tissue of aging mice. A comparison of the results illustrated in Supplementary Figures S2H–J revealed a remarkable increase in the levels of IL-6, TNF-α and IL-1β in the brains of D-gal-induced aging mice. Furthermore, after UB treatment, the activities of these inflammatory cytokines were significantly inhibited [IL-6: *F* (4, 25) = 34.91, *p* < 0.01; TNF-α: *F* (4, 25) = 74.22, *p* < 0.01; IL-1β: *F* (4, 25) = 99.84, *p* < 0.01]. In brief, these data indicated reduced D-gal-induced production of proinflammatory cytokines.

#### Effects of UB on Hippocampal Pathology in Aging Mice

**FIGURE 4:**
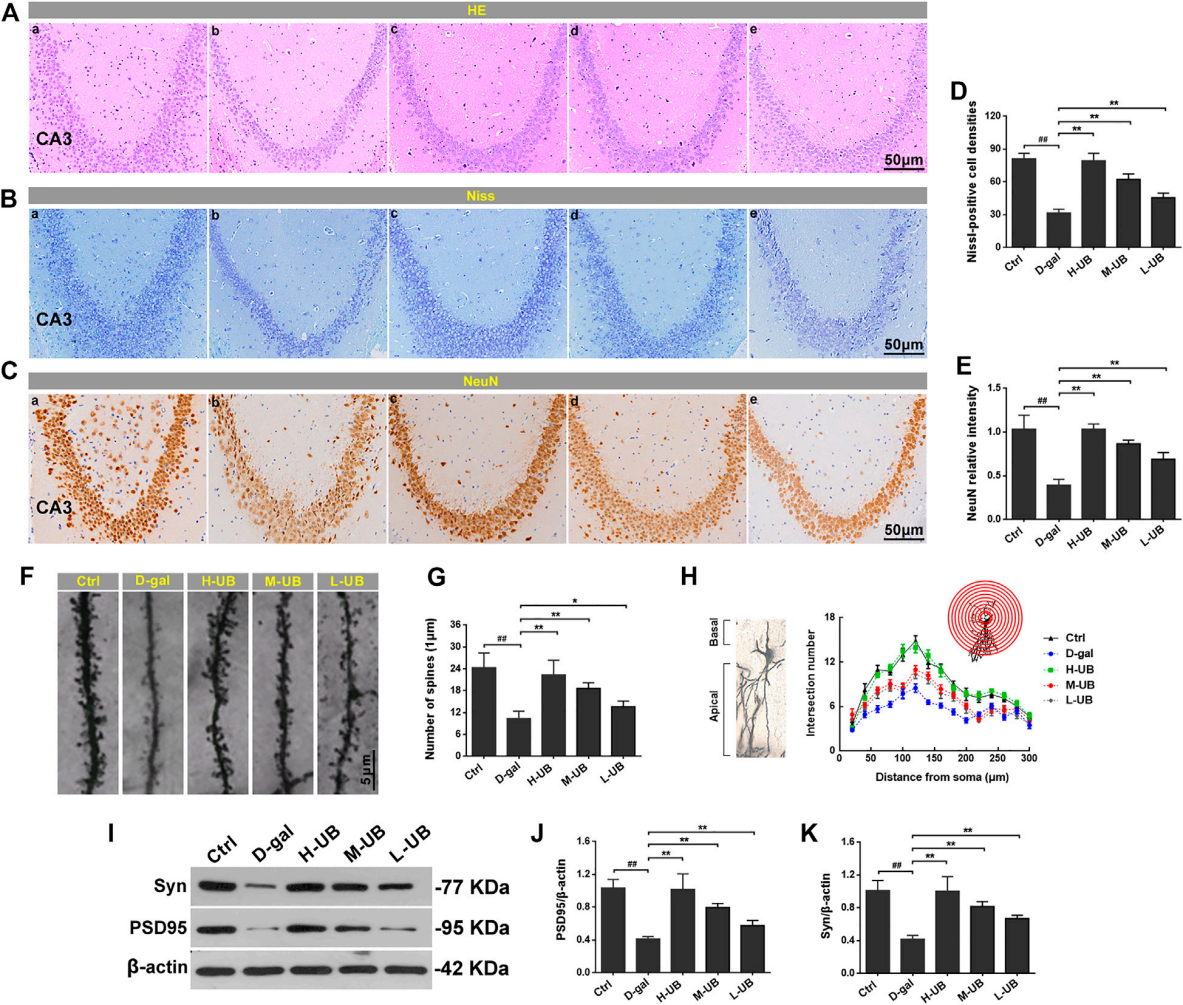
Effect of UB on hippocampal pathology in D-gal-treated aging mice. Representative H&E **(A)** and Nissl **(B)** staining of hippocampal alterations. **(C)** Immunohistochemistry of NeuN in the hippocampus of each group. **(D)** The Nissl-positive cell densities for each group was counted at high magnification (*n* = 6 per group). **(E)** The statistical analysis of NeuN relative intensity (*n* = 6 per group). Representative high magnification images **(F)** and quantification of the spine number in aging mice **(G)** (*n* = 6 per group). **(H)** At least 10 neurons per group were analyzed by Sholl analysis. **(I)** The expression of Syn and PSD95 was detected by western blotting. Quantification analysis of PSD95 **(J)** and Syn **(K)**. Data were normalized to β-actin protein expression (*n* = 6 per group). All results are described as means ± SD. ^#^ *p* < 0.05 and ^##^ *p* < 0.01 vs the control group; ^*^ *p* < 0.05 and ^**^ *p* < 0.01 vs the D-gal-induced aging group.

Previous studies of rodents have reported that the hippocampus is a region of the brain that plays a significant role in learning, memory and cognition, so histological evaluation of the brain hippocampus of mice was conducted using HE and Nissl to examine whether UB could exert beneficial effects against D-gal-induced brain damage in aging mice. According to our findings, the histological features in the CA3 region of the mouse hippocampus revealed no remarkable hippocampal neuronal abnormalities in healthy control mice. Compared with aging model mice induced by D-gal, severe structural damage was characterized by HE staining of disorganized nerve fibers with irregular neurons and apoptosis-like cells (Figure 4A). However, these pathological changes were obviously reversed after UB administration, and the high-dose UB group (150 mg/kg) exerted a maximum protective effect (only minor alterations were observed in the morphological structure), which was almost identical to the results obtained for the normal control groups. Additionally, we also observed changes in the cortex, which were consistent with the hippocampus.

The changes in Nissl bodies were also investigated by Nissl staining, and significant changes in the decreased amounts of the CA3 region Nissl bodies were found in the aging mice. However, after treatment with UB, the number of neurons in the hippocampus was obviously recovered [*F* (4, 25) = 53.43, *p* < 0.01; Figures 4B,D]. Furthermore, we also observed the expression of NeuN in aging mice through immunohistochemical methods. The results showed a notable decrease in the number of NeuN-immunostained neurons in aging mice when compared to those in the control group [*F* (4, 25) = 68.17, *p* < 0.01; Figures 4C,E]. However, the number of NeuN-immunostained neurons in UB-treated mice was significantly increased compared with that in D-gal-induced aging animals.

Additionally, to further verify the effect of UB on the structural plasticity induced by D-gal, the alterations in spine density in the hippocampal region of the brain were detected using Golgi staining. The number of neuronal spines was significantly decreased in aging compared with control mice, while preintervention with UB alleviated this decrease (all *p* < 0.01, Figures 4F,G). By concentric circle (Sholl) analysis (Figure 4H), we found that the neurite arborization was significantly reduced in the aging mice compared with the control mice, whereas pretreatment with UB alleviated this decrease. At the molecular level, expression of the postsynaptic marker PSD95 and the presynaptic marker synapsin І in hippocampal tissue was inspected through WB. As shown in Figures 4I–K, intervention with D-gal in aging mice markedly reduced the expression of PSD95 and synapsin І, while the levels of these proteins were both restored by pretreatment with UB [PSD95: *F* (4, 10) = 120.76, *p* < 0.01; synapsin І: *F* (4, 10) = 171.45, *p* < 0.01]. In summary, UB can exert a protective effect in improving hippocampal pathology in aging mice.

#### Regulatory Effects of UB in Inhibiting the Activation of Microglia and Astrocytes in Aging Mice

Then, to further investigate the effect of UB on the activation of glial cells such as astrocytes and microglia, immunohistochemical staining for GFAP and IBA1 (markers of astrocytes and microglia) was performed in aging mice. As shown in Supplementary Figures S3C–F, D-gal significantly increased the number of GFAP-immunoreactive astrocytes and IBA1-immunoreactive microglia compared with the control group [GFAP: *F* (4, 25) = 59.62, *p* < 0.01; IBA 1: *F* (4, 25) = 84.85, *p* < 0.01], while UB treatment decreased these differences. Furthermore, we also verified the above results using WB, and as shown in Supplementary Figures S3G–I, the expression of GFAP and IBA1 was significantly increased compared with that in controls after D-gal treatment (all *p* < 0.01). However, after administration of UB to aging mice, GFAP and IBA1 expression levels were significantly reduced, suggesting that UB prominently inhibited the activation of glial cells induced by D-gal in mice.

#### UB Supplementation Ameliorates Advanced Glycation in Aging Mice

**FIGURE 5:**
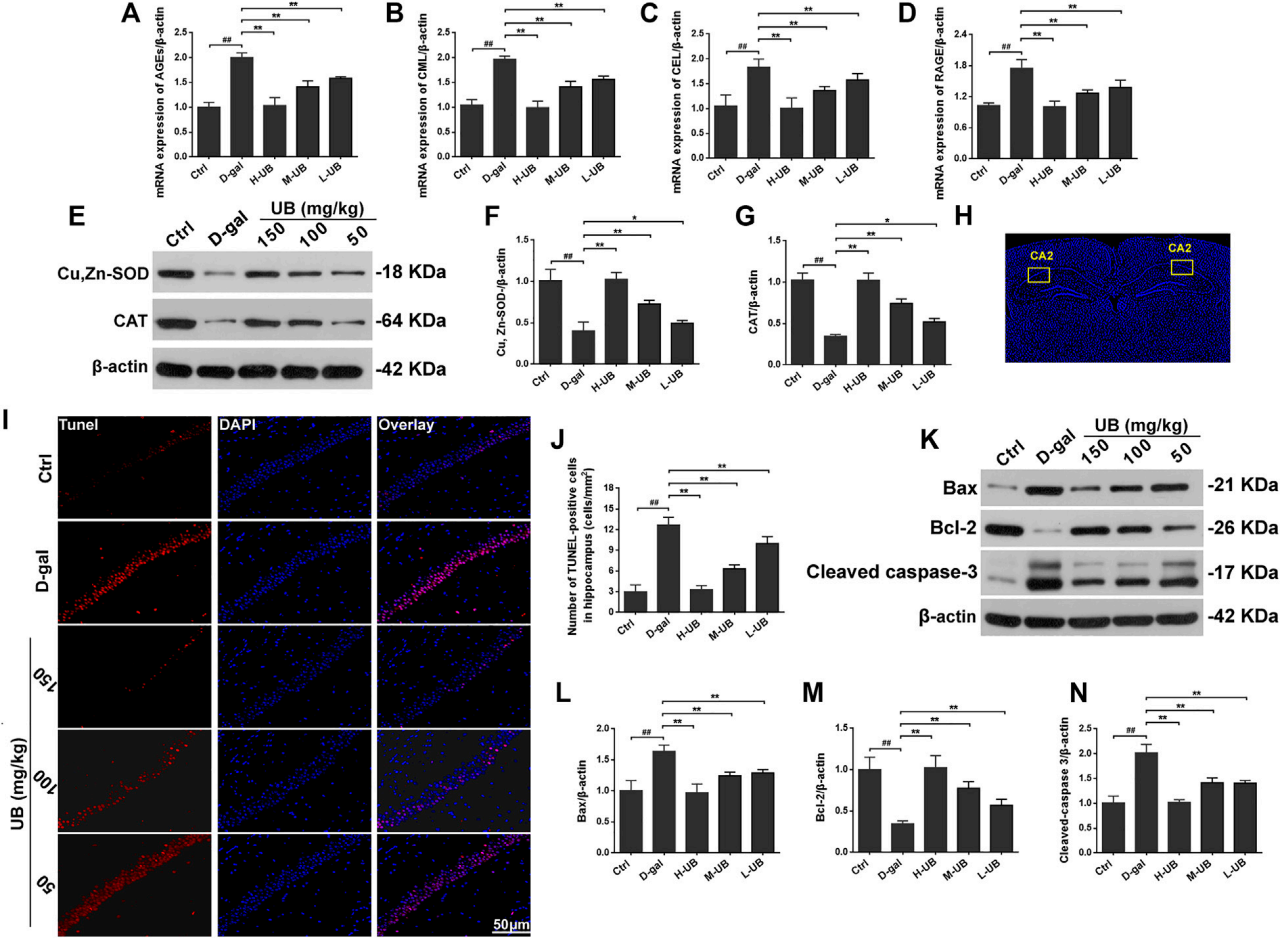
UB supplementation ameliorated the mRNA expression of AGEs **(A)**, CML **(B)**, CEL **(C)** and RAGE **(D)** in D-gal-induced aging mice (*n* = 6 per group). **(E)** UB enhanced the activity of Cu, Zn-SOD and CAT in hippocampal tissue. Quantification analysis of Cu, Zn-SOD **(F)** and CAT **(G)**. Data were normalized to β-actin protein expression (*n* = 6 per group). **(H)** Representative tunel staining of the hippocampal CA2 region. **(I)** TUNEL assays were performed to evaluate apoptosis in the hippocampus *in vivo.* **(J)** The relative proportion of TUNEL-positive cells in the hippocampus of aging mice (*n* = 6 per group). **(K)** Protein expression of Bax, Bcl-2 and cleaved caspase-3 in the brains of aging mice was detected by western blotting. Quantification analysis of Bax **(L)**, Bcl-2 **(M)** and cleaved caspase-3 **(N)**. Data were normalized to β-actin protein expression (*n* = 6 per group). All results are described as means ± SD. ^#^ *p* < 0.05 and ^##^ *p* < 0.01 vs the control group; ^*^ *p* < 0.05 and ^**^ *p* < 0.01 vs the D-gal-induced aging group.

The state of advanced glycation is generally assessed using AGEs, including CML and CEL or RAGE. RT-PCR was used to examine the formation of AGEs, CML and CEL in groups of mouse brains, and the data showed that D-gal-administered mice had higher mRNA levels of AGEs, CML and CEL than mice in the control group (all *p* < 0.001, Figures 5A–C). Interestingly, the formation of AGEs, CML and CEL in the brains of D-gal-induced aging mice was significantly reduced after UB intervention [AGEs: *F* (4, 10) = 24.75, *p* < 0.01; CML: *F* (4, 10) = 94.36, *p* < 0.01; CEL: *F* (4, 10) = 21.18, *p* < 0.01]. Furthermore, as a multiligand receptor belonging to the immunoglobulin superfamily of cell surface molecules acting as a counterreceptor for diverse molecules, RAGE expression in mRNA was also analyzed. As shown in Figure 5D, the expression of RAGE was obviously increased in D-gal-treated aging mice, while it was prominently reduced with the administration of UB.

#### UB Downregulates the Enzyme Activity of Cu/ZnSOD and CAT in the Brain Tissue of Aging Mice

Previous literature has reported that age-related changes are accompanied by alterations in redox homeostasis and oxidative stress (Chen et al., 2021). We further confirmed that the antioxidative effect of UB was beneficial for the improvement of cognitive and memory function behavior in the same animals. The enzyme activity of Cu/ZnSOD and CAT was analyzed by western blotting in this study. As shown in Figures 5E–G, compared with the control group, intraperitoneal injection of D-gal at a dose of 150 mg/kg/d significantly decreased the expression of Cu, Zn-SOD and CAT [Cu, Zn-SOD: *F* (4, 10) = 130.34, *p* < 0.01; CAT: *F* (4, 10) = 75.79, *p* < 0.01]. However, in the group treated with D-gal plus UB, the expression levels of Cu, Zn-SOD and CAT were dramatically higher than those in D-gal-treated aging mice. Therefore, the above data suggested that UB administration could promote the activities of these antioxidant enzymes in the brain tissues of D-gal-induced aging mice to ease the aging process.

#### UB Inhibits D-Gal-Induced Hippocampal Neuron Apoptosis

Next, we evaluated the effect of UB on apoptosis in D-gal-induced damaged hippocampal neurons. The results of the TUNEL assay revealed that the total number of TUNEL-positive cells was remarkably scattered in the hippocampus compared with normal control mice [*F* (4, 25) = 66.54, *p* < 0.01; Figures 5I,J]. However, there were fewer TUNEL-positive neurons in the hippocampus of the D-gal-induced aging mice co-treated with UB compared with the mice treated with D-gal (*p* < 0.01).

Moreover, using western blotting, we also inspected the influence of apoptosis on the neuroprotection of UB, and we investigated the expression of Bax and Bcl-2, as well as the cleavage and activation of caspase-3, in D-gal-induced aging mouse brains. In the D-gal-induced aging mouse group, a significant increase in Bax and a decrease in Bcl-2 were observed [Bax: *F* (4, 25) = 142.41, *p* < 0.01; Bcl-2: *F* (4, 25) = 42.07, *p* < 0.01; Figures 5K–N], while after treatment with UB at doses from 50 to 150 mg/kg, the expression of Bax was decreased and the expression of Bcl-2 was increased, indicating its neuroprotective properties. As we have previously reported, D-gal could activate caspase-3 in the brains of mice. In the current study, activation of caspase-3 in D-gal-treated aging mice could be significantly suppressed through UB intervention. The above data illustrated the establishment of a brain aging model, whereas UB administration exerted a neuroprotective effect on hippocampal damage associated with D-gal-induced brain aging.

#### UB Suppressed JNK Activation and Prevented Cytochrome c Release in Aging Mice

**FIGURE 6:**
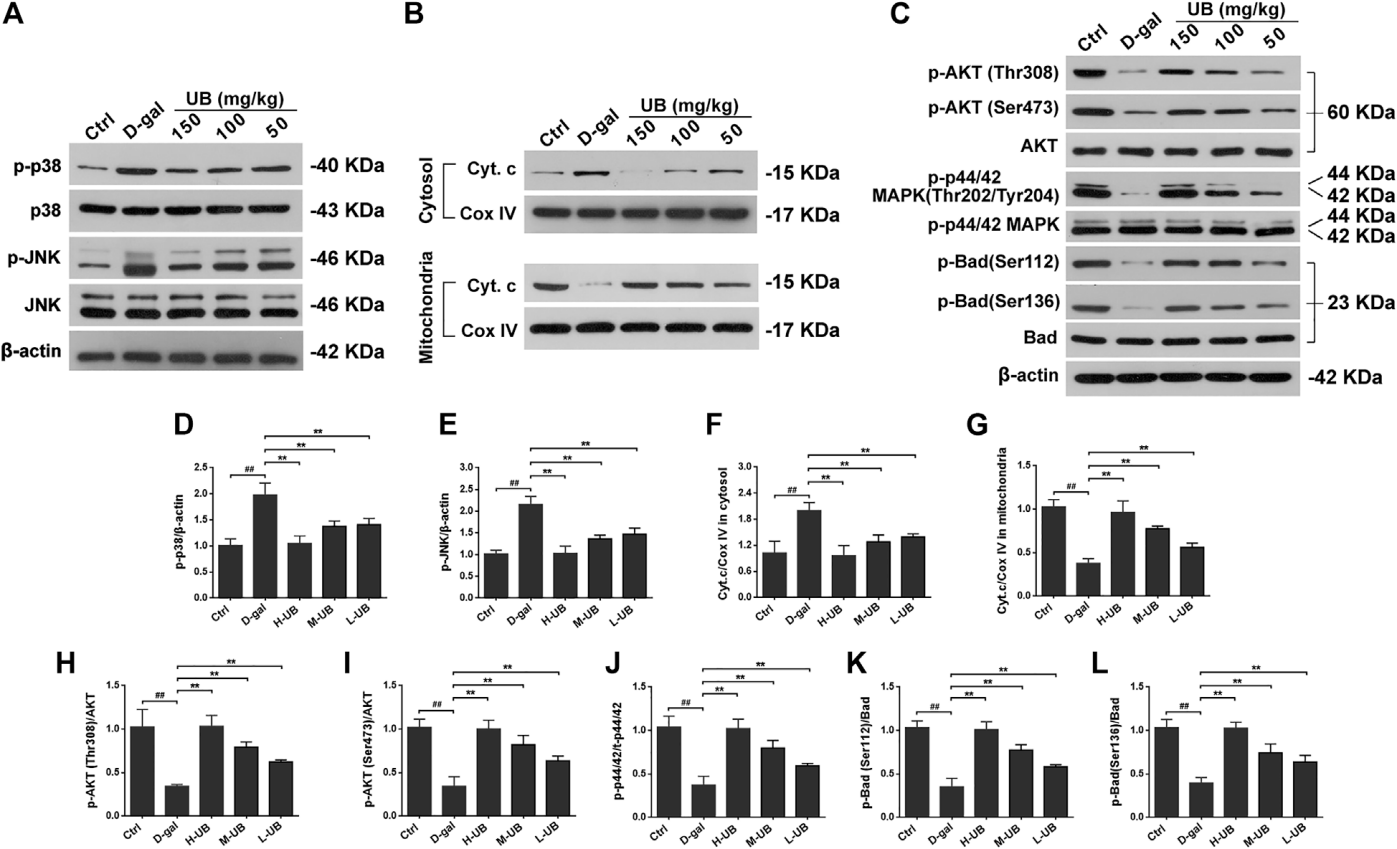
**(A)** Western blots and densitometric analysis of the phosphorylation of stress-activated protein kinase (SAPK)/c-Jun NH 2-terminal kinase (JNK) and p38 MAPK in the brains of aging mice. **(B)** Western blots and densitometric analysis of cytosolic and mitochondrial Cyt C and COX IV in all treated groups. **(C)** Western blots and densitometric analysis of the phosphorylation of Akt, p44/42 MAPK and Bad in the brains of D-gal-treated aging mice. Quantification analysis of p-p38 **(D)**, p-JNK **(E)**, Cyt.c/COX IV in cytosolic **(F)** and mitochondrial **(G)**, p-Akt (Thr308)/Akt **(H)**, p-Akt (Ser473)/Akt **(I)**, p-p44/42/t-p44/42 **(J)**, p-Bad (Ser112)/Bad **(K)** and p-Bad (Ser136)/Bad **(L)**. Data were normalized to β-actin protein expression (*n* = 6 per group). All results are described as means ± SD. ^#^ *p* < 0.05 and ^##^ *p* < 0.01 vs the control group; ^*^ *p* < 0.05 and ^**^ *p* < 0.01 vs the D-gal-induced aging group.

The relative expression of JNK/p38 MAPK protein was measured via western blotting in the present study. The protein expression of p-JNK and p-p38 MAPK was lower in the control group than in the brains of D-gal-induced aging mice [p-JNK: *F* (4, 25) = 36.21, *p* < 0.01; p-p38 MAPK: *F* (4, 25) = 91.65, *p* < 0.01; Figures 6A,D,E]. However, the group cotreated with UB and D-gal displayed notably higher relative protein expression levels of p-JNK and p-p38 MAPK than the mice treated with D-gal (all *p* < 0.01).

Activation of the p38/JNK pathway is thought to be responsible for cytochrome c (Cyt C) release, which results in the activation of effector caspase-3 and causes apoptosis. In the current research, the expression of mitochondrial and cytosolic Cyt C in the experimental mouse brains was analyzed by western blotting. In mice without D-gal administration, the expression of Cyt C was rarely detectable in the cytoplasm in the brains of aging mice. It is worth noting that the intensities of Cyt C in mitochondria in the brains of D-gal-treated groups were significantly decreased compared with those in control mice and were upregulated in the groups treated with the combination of D-gal and UB (all *p* < 0.01, Figures 6B,F,G). Moreover, there was a corresponding decrease in the activity and release of Cyt C from mitochondria into the cytosol after UB treatment (150, 100, 50 mg/kg) in aging mice.

#### UB Promotes the Phosphorylation of Akt and p44/42 MAPK in the Mouse Brain of D-Gal-Treated Aging Mice

Akt and p44/42 MAPK (ERK1/2) are a widely conserved family of serine/threonine protein kinases that can participate in endogenous activities, such as cell migration, proliferative division and survival. To verify the regulatory effects of UB on the expression of Akt and the underlying p44/42 MAPK signaling pathway mechanism, the effects of UB on these pathways were investigated using western blot analysis. Phosphorylation is always the best studied posttranslational modification of AKT, and when both threonine 308 (Thr308) and serine 473 (Ser473) residues are phosphorylated, Akt kinase can be fully activated; thus, we studied the level of phosphorylation at both Thr308 and Ser473. As demonstrated in Figures 6C,H,I, the phosphorylation status of Thr308 and Ser473 of Akt in aging model mice was significantly increased compared with that in the control group [Akt (Thr308): *F* (4, 25) = 29.52, *p* < 0.01; Akt (Ser473): *F* (4, 25) = 39.44, *p* < 0.01]. In addition, the decrease in pAkt (Thr308/Ser473) in aging model mice was dramatically attenuated by UB intervention (all *p* < 0.01).

We also analyzed the activation status of p44/42 MAPK by western blotting. Consistent with previously published reports, treatment of animals with D-gal dramatically suppressed the phosphorylation of p44/42 MAPK (Figures 6C,J). Additionally, UB markedly increased the phosphorylation (activation) of p44/42 MAPK in D-gal-induced aging mice.

#### Effects of UB on the Phosphorylation of Bad in the Brains of D-Gal-Treated Aging Mice

In the current study, we further investigated the role of Bad, a proapoptotic protein of the BCL2 family, in D-gal-mediated cell apoptosis. Previous studies have shown that Bad is modulated, requiring the phosphorylation of two regulatory serine residues (serine 112 (Ser-112) and serine 136 (Ser-136)), so we analyzed total Bad and phospho-Bad (Ser112, Ser136) expression by western blotting. The phosphorylation levels of Bad at Ser112 and Ser136 were significantly decreased in D-gal-induced brain aging mice. As expected, phosphorylation at sites Ser112 and Ser136 was significantly increased in UB-treated aging mice, which could be associated with the regulation of neuronal survival by promoting Bad phosphorylation [pBad (Ser112): *F* (4, 25) = 114.37, *p* < 0.01; pBad (Ser136): *F* (4, 25) = 141.28, *p* < 0.01; Figures 6C,K,L]. These data strongly suggested that the regulation of Bad phosphorylation played an active role in mediating *D*-gal-induced apoptosis of neuronal cells.

#### Effect of UB on Normal Young Mice (2 months Old) and Naturally Senile Mice (12 months Old)

It has been confirmed that the ameliorative effect of UB is involved in inhibiting Cyt C-mediated apoptosis and promoting the survival of neurons *via* the PI3K pathway in D-gal-induced aging mice; however, the mechanism of UB in normal young mice and naturally aging mice remains unclear. Thus, 2-month-old (normal young) and 12-month-old (naturally aging) mice received UB treatment for 2 months, and the corresponding protein expression was detected in the current study.

**FIGURE 7:**
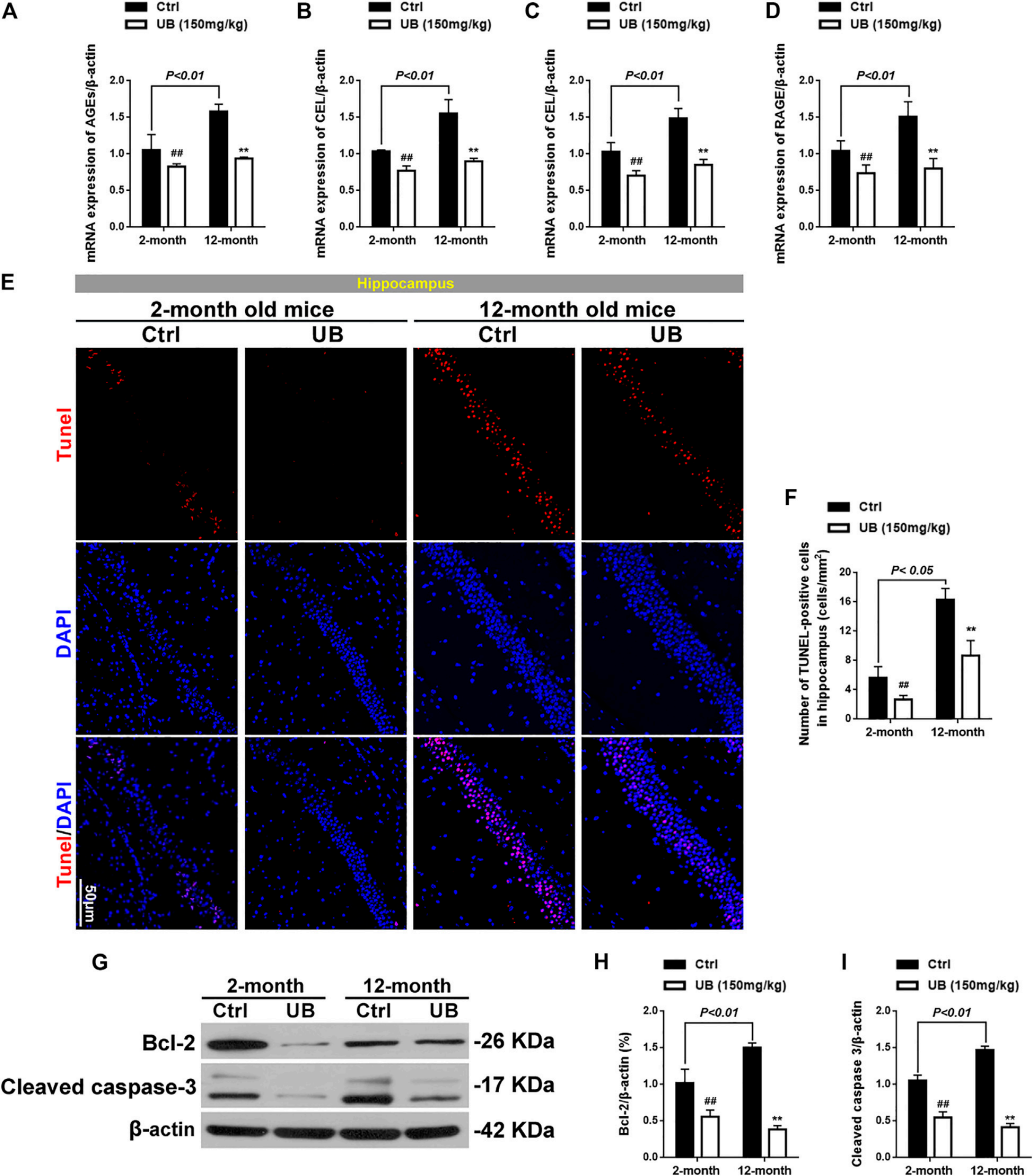
Expression of AGEs **(A)**, CML **(B)**, CEL **(C)** and RAGE **(D)** in 2-months- and 12-months-old mice by quantitative RT-PCR (*n* = 6 per group). **(E)** TUNEL assays were performed to evaluate apoptosis in the hippocampus of normal young and naturally senile mice *in vivo.* **(F)** The relative proportion of TUNEL-positive cells in the hippocampus of normal young and naturally senile mice (*n* = 6 per group). **(G)** Protein expression of Bcl-2 and cleaved caspase-3 in the brains of normal young and naturally senile mice was detected by western blotting. Quantification analysis of Bcl-2 **(H)** and cleaved caspase-3 **(I)**. β-actin served as an internal control (*n* = 6 per group). All results are described as means ± SD. ^#^ *p* < 0.05 and ^##^ *p* < 0.01 vs the control group; ^*^ *p* < 0.05 and ^**^ *p* < 0.01 vs the 12-months-old aging group.

**FIGURE 8:**
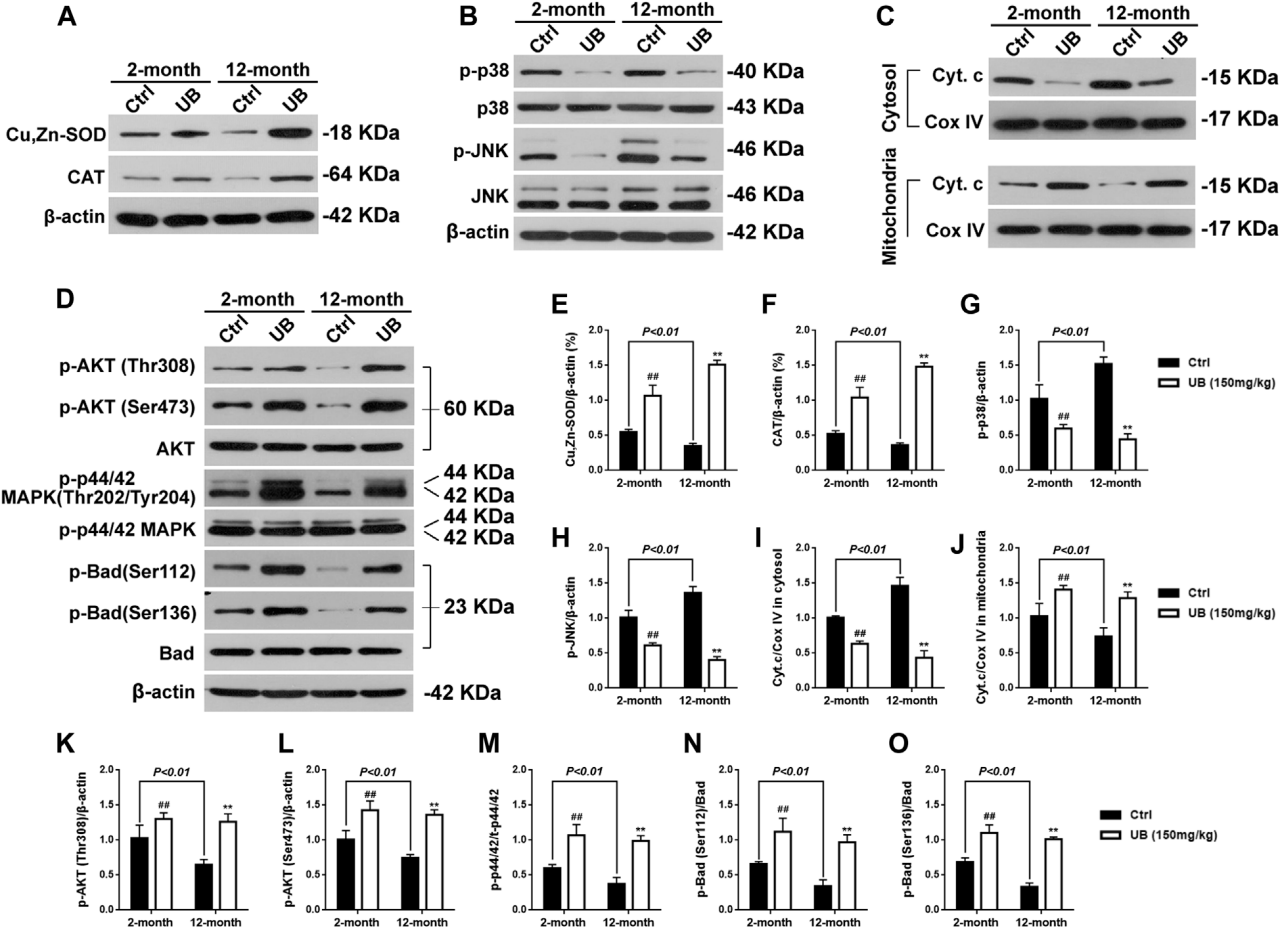
Underlying molecular mechanisms of UB in normal young mice and naturally senile mice. **(A)** UB enhanced the activity of Cu, Zn-SOD and CAT in hippocampal tissue of 2-months- and 12-months-old mice. **(B)** UB suppressed the activation of p-JNK and p-p38 in hippocampal tissue of 2-months- and 12-month-old mice. **(C)** UB inhibited the release of Cyt C from mitochondria in the brains of 2-months- and 12-month-old mice. **(D)** UB facilitated the phosphorylation of Akt, p44/42 MAPK and Bad in the brains of 2-months- and 12-months-old mice. Quantification analysis of Cu, Zn-SOD **(E)**, CAT **(F)**, p-p38 **(G)**, p-JNK **(H)**, Cyt.c/COX IV in cytosolic **(I)** and mitochondrial **(J)**, p-Akt (Thr308)/Akt **(K)**, p-Akt (Ser473)/Akt **(L)**, p-p44/42/t-p44/42 **(M)**, p-Bad (Ser112)/Bad **(N)** and p-Bad (Ser136)/Bad **(O)**. β-actin served as an internal control (*n* = 6 per group). All results are described as means ± SD. ^#^ *p* < 0.05 and ^##^ *p* < 0.01 vs the control group; ^*^ *p* < 0.05 and ^**^ *p* < 0.01 vs the 12-months-old aging group.

As shown in Figures 7A–D, the mRNA expression of AGEs, CML, CEL and RAGE was significantly increased in 12-month-old mice compared with 2-month-old normal young mice [AGEs: *F* (3, 20) = 76.43, *p* < 0.01; CML: *F* (3, 20) = 82.39, *p* < 0.01; CEL: *F* (3, 20) = 43.16, *p* < 0.01; RAGE: *F* (3, 20) = 55.64, *p* < 0.01]. Furthermore, among both normal young mice and naturally senile mice treated with UB for 2 months, all groups revealed a decrease in the mRNA levels of these proteins. Expression of antioxidant enzymes such as Cu, Zn-SOD and CAT in 12-month-old aging mice was significantly decreased in comparison that in 2-months-old normal young mice (all *p* < 0.01, Figures 8A,E,F). Furthermore, among both normal young mice and naturally senile mice treated with UB for 2 months, all groups revealed an increase in the expression of these proteins. Importantly, close attention should be paid to the significant increase in Cyt C in mitochondria in 12-month-old aging mice, as well as the markedly abolished accumulation of cytosolic Cyt C in response to UB both in 2-month-old young mice and 12-months-old aging mice (*p* < 0.05 or *p* < 0.01, Figures 8C,I,J). Similar to the results obtained for aging mouse models based on the administration of D-gal, UB downregulated JNK/p38 (Figures 8B,G,H) and upregulated PI3K/Akt (Figures 8D,K–M), improved cognitive deficits (Supplementary Figures S4A–E), ameliorated structural plasticity (Supplementary Figures S4F–K), suppressed apoptosis (Figures 7E–I), and attenuated astrocyte and microglial activation (Supplementary Figure S5) in hippocampal tissue of normal aging mice.

## Discussion

Aging happens to all of us, is generally thought of as a natural part of life, and exhibits neurologic symptoms or functional changes, including memory and cognitive impairment (Schöll et al., 2016; Ikram et al., 2021). Compounds from natural sources in modern drug discovery against age-related ailments tend to be an efficient rehabilitation intervention and an effective treatment to delay brain aging (Mozaffarian and Wu, 2018). At present, prevention of aging by promoting resistance to oxidative stress, inflammation and apoptosis is a hot spot of research. In our research, we first evaluated the antioxidant properties of UB *in vitro* using DPPH, ABTS^**+**^, and O_2_^**.**^ and ^**.**^OH assays*.* Then, we assessed the neuroprotective activity of UB on H_2_O_2_-induced OS injury and apoptosis in neuro2a cells. Furthermore, in an *in vivo* animal model of brain aging, administration of D-gal with subcutaneous injection at a dose of 150 mg/kg/d to mice for 8 weeks was performed to investigate the action of the most effective fraction on AD-like symptoms of gradually impaired memory and cognitive capacity. To the best of our knowledge, the present study demonstrated for the first time that 1) UB possessed good antioxidant activity in a free radical-based assay; 2) UB sufficiently improved cell apoptosis induced by H_2_O_2_ *in vitro*; and 3) UB efficiently protected brain aging mice from D-gal-induced oxidative injury. Additionally, the function of UB was correlated with the PI3K pathway and inhibited Cyt-C-mediated apoptosis.

OS theory is one of most popular hypotheses accelerating aging. Brain tissues are particularly vulnerable to deteriorated age-related redox homeostasis (Roger-Sánchez et al., 2012). UB, the gut metabolite of ETs and a class of antioxidant polyphenols, was protective against OS in multiple organs, such as the intestinal tract, liver and kidney (Yuan et al., 2015; Savi et al., 2018). Based on the free radical theory of aging, we detected the effect of UB on OS status during the progression of brain aging, as revealed by decreased GSH-Px levels, SOD, CAT, and T-AOC activities and increased MDA content. Under the situation of OS, SOD is the first security force in the antioxidant defense system, and under its action, four-electron reduction of O.- to H_2_O_2_ occurs, after which H_2_O_2_ is mainly further eliminated through catalysis by CAT and GSH-Px, directly leading to the production of H_2_O and O_2_ (Sikandar et al., 2019). T-AOC is representative of comprehensive antioxidant enzymes in various tissues of the body, and MDA is commonly used as a biomarker of oxidative stress, which is a lipid oxidation final product (Wu et al., 2020a). In accordance with previous reports, anomalous changes in the activities of enzymatic and nonenzymatic antioxidants due to D-gal exposure were found in the current study, and our experimental findings showed that UB intervention could distinctly attenuate the MDA content and antioxidant enzyme activities in mice, which may be one mechanism by which UB improves learning and memory in D-gal-exposed aging mice (Lu et al., 2010; Savi et al., 2018). Interestingly, it has been reported that lipid peroxidation can cause ferroptosis, and aging-associated cognitive dysfunction is closely related to ferroptosis of neurons. At present, it is not clear whether ferroptosis exists in D-gal-induced brain aging; therefore, OS and lipid peroxidation are still hot topics for further study. Overall, the above data suggested that UB had antiaging activity and could protect the brain tissue of mice from D-gal-induced oxidative damage.

Recently, several studies have shown that glial cell activation, such as astrocytes and microglia, seems to correlate with misfolding and abnormal Aβ accumulation in early-onset AD and in animal AD models exposed to Aβ or D-gal (Chen et al., 2019; Wu et al., 2019). The findings presented herein are consistent with what has been found in previous studies showing that cognitive dysfunction caused by long-term administration of D-gal leads to increased levels of IBA1 (a marker for microglia) and GFAP (a marker for astrocytes) in aging mice, suggesting that neuroinflammation and the accompanying activation of glial cells such as astrocytes and microglia may contribute to age-related cognitive decline, including synaptic dysfunction manifested in an age-related manner (Harach et al., 2017). Interestingly, UB can utilize anti-inflammatory and antioxidant functions to drive its neuroprotective capabilities to inhibit the activation of astrocytes and microglia in D-gal-induced neurotoxicity in aging mice. Furthermore, relieving glial cell activation caused by AD and AD-associated NDs has become a hot spot of current research, and scholars have proposed many different phenotypes of glial cells (such as A1/A2 astrocytes, disease-associated microglia (DAM), and lipid droplet-accumulated microglia (LDAM)) (Marschallinger et al., 2020). Thus, we anticipate further reports on the effect of UB on the phenotypic transformation of these cells in neurodegenerative diseases.

At high concentrations, ROS can induce oxidative damage and contribute to neuronal cell death, occurring whenever they are generated in the context of an apoptotic process (Xu et al., 2019). The TUNEL assay is programmed to estimate the number of cells with DNA damage, termed TUNEL-positive cells *in situ* labeling of apoptosis-induced DNA strand breaks (Takeda et al., 2015). As products of the gut microbiota metabolism of EA, urolithins have been reported to contribute to the anti-inflammatory and neuroprotective effects of EA, and their potential anti-apoptotic properties have recently been a topic of interest. Previous studies have revealed that UA and UB and their methylated derivatives mitigate apoptosis and caspase 3/7 and 9 release from the OS of BV-2 and SH-SY5Y cells *in vitro* (Xu et al., 2018; Lee et al., 2019). It is worth noting that the data from animal experiments *in vivo* showed that D-gal significantly promoted an increase in the total number of TUNEL-positive cells in the hippocampus and cerebral cortex during the brain aging process. In addition, our study demonstrated that D-gal induced the apoptosis pathway by promoting the activation of caspase-3. Interestingly, we observed that the number of TUNEL-positive cells declined after UB administration to animals, and activation of caspase-3 was also inhibited in D-gal-exposed aging model mice. There, it is not hard to surmise that UB was able to significantly reduce the overall OS level throughout the aging process, which might contribute to the protective effect of UB in cell apoptosis.

MAPKs participate in the regulation of a broad range of crucial cellular processes, including cell migration, differentiation, proliferation and apoptosis (Liu et al., 2012). JNK, also known as stress-activated protein kinase (SAPK) and p38, are activated by environmental stresses such as OS, and signaling pathways participate in mediating apoptosis in host cells upon injury or infection (Kaneto et al., 2004). Furthermore, the presence and activation of JNK/p38 effectively promotes Cyt-C release from mitochondria into the cytosol, and JNK/p38-mediated Cyt-C release contributes to an increase in the activity of caspase-3 concomitantly with the onset of apoptosis (Karaosmanolu, 2020). Furthermore, the activation of poly (ADP-ribose) polymerase 1 (PARP-1) in response to upregulation of p-JNK levels is another symbol of neuronal apoptosis (Johnson and Nakamura, 2007). In contrast, using specific small-molecule compounds targeting a p-JNK-like JNK inhibitor (SP600125) and peptide or natural medicine alleviates JNK-mediated neurodegeneration and neuroapoptosis. Treatment with D-gal significantly enhanced JNK/p38 expression and activated the release of Cyt-C from mitochondria into the cytosol. In the present study, we found that D-gal exposure in mice led to the activation of JNK/p38 and cytochrome c release, and this action was evidently blocked through UB treatment. In addition, it has been shown that when neurons are exposed to damage or pathogen-associated proteins/markers from adjacent cells, such as microglia and astrocytes, external pathways that initiate apoptosis are activated directly (Harach et al., 2017). Consistently, UB can effectively inhibit the activation and expression of GFAP and IBA1 in the brain tissues of D-gal-induced aging mice to achieve the effective neuroprotective function of UB therapy. Taken together, UB targeting of apoptosis-related proteins such as Cyt-c, Bax and Caspase-3 to inhibit cell apoptosis may be a potential approach to improve neuronal resistance in brain tissues associated with age-related diseases.

Numerous lines of evidence have shown that a pro-apoptotic member of the Bcl-2 family, the protein Bad, plays a significant effect in the regulation of cell death/survival (Wu et al., 2020b). Thus, bad may be an integrated signaling molecule (converging checkpoint) between the surviving PI3-K/Akt pathway and the JNK-mediated death pathway in neurons (Huang, 2015). The phosphorylation of Bad (Ser136) is mediated by Akt and then binds with 14-3-3 and prevents Bad from attaching to Bcl-XL (Donovan et al., 2002). In contrast, the affinity of Bad for 14-3-3 is reduced by JNK, and the association of Bad with Bcl-XL or Bcl-2 via phosphorylation of Bad (Ser128) or 14-3-3 is also promoted. Bad has been widely reported to be involved in neuronal cell death during brain aging (Sunayama et al., 2005). Recently, some neuroprotective agents or cell death inhibitors that target only cell signaling pathways have been unsuccessful. Elucidating the molecular signaling mechanism of the integration of survival and apoptosis signals is the key to regulating cell death/survival (Härdtner et al., 2020). Bad may act as a molecular switch for both survival and apoptotic signals to maintain cell and tissue homeostasis and may contribute to cell fate (Donovan et al., 2002; Wu et al., 2020b).

High expression levels of PI3K and AKT induce the phosphorylation of Bad, thereby participating in the suppression of oxidative stress-induced cell death and promotion of cell survival related to neurodegenerative disorders including AD and PD (Long et al., 2021; Yoon et al., 2021). Akt phosphorylates Bad at Ser136 to promote cell survival, and phosphorylation of Bad at Ser136 is mediated by the serine/threonine protein kinase Akt (Akt is a potent Bad Ser136 kinase *in vitro*). In neurons of the brains of aging mice exposed to D-gal, we found that AKT phosphorylation at Ser473 and Thr308 was transiently decreased along with a downregulation of phosphorylation at Ser136 (Donovan et al., 2002; Wang et al., 2021). Statistics indicated that the phosphorylation of Bad (Ser136), which is related to the phosphorylation of Akt (Thr308/Ser473), was vitally upregulated by UB in D-gal-induced aging mice. Therefore, activating the PI3K signaling pathway was considered to be a profitable tactic to treat aging in response to oxidative injury.

The p44 and p42 MAP kinases (p44/p42), also called ERK1/2, have been shown to be critical regulators of cell differentiation, cell physiology and neuronal function through phosphorylation of Bad at Ser112 (Patel et al., 2016). Consistent with the results reported above, the present study revealed that basal phosphorylation of Bad-Ser112 was decreased in aging mice induced by D-gal accompanied by the inhibition of phosphorylation of p44/42 MAPK at Thr202 and Tyr204 (Margadant et al., 2013). Importantly, UB administration markedly decreased the phosphorylation and activation of p44/42 MAPK in D-gal-exposed aging model mice, which led to the induction of survival factor signaling that stimulated the phosphorylation of Bad at multiple sites, including Ser-136 and Ser-112. Phosphorylation of these two sites by several kinases, including Akt, has been associated with the promotion of Bad binding to 14-3-3 protein, which may sequester Bad from Bcl-XL or Bcl-2, thus promoting and maintaining neuronal survival (Le Mercier et al., 2021).

## Conclusion

In summary, experimental data elucidated that chronic exposure to D-gal induces multiple molecular, cellular, structural, and functional changes in a mouse model of brain aging in parallel with simulated symptoms of natural senescence. In D-gal-treated aging mice, pretreatment with UB succeeded in promoting neuronal survival by protecting against OS and ultimately led to an amelioration of neurological deficits and cognitive performance in aging mice. The underlying molecular mechanisms might occur through concomitant phosphorylation of the proapoptotic protein Bad at Ser-136 and Ser-112 via simultaneous activation of the PI3K/Akt pathways and ERK pathway to promote neuronal survival and ameliorate the learning and memory impairment. Thus, UB may be considered an effective supplementation to avoid OS-induced memory impairment and brain injury.

## Data Availability

The datasets presented in this study can be found in online repositories. The names of the repository/repositories and accession number(s) can be found in the article/Supplementary Material.

## Supplementary Material

Supplementary Figure 1 The *in vitro* antioxidant/antiradical activity of UB was determined using four systems: **(A)** ABTS^+^ radical scavenging assay; **(B)** DPPH radical scavenging assay; **(C)** OH^–^ radical scavenging assay; **(D)** O^2^ radical scavenging assay. All results are described as means ± SD (*n* = 6 per group).

Supplementary Figure 2 Effect of UB on Ach-E **(A)** and MAO **(B)** activities in brain tissue of aging mice. Effect of UB on the activities of CAT **(C)**, GSH-Px **(D)**, SOD **(E)**, T-AOC **(F)** and MDA levels **(G)** in the brain tissue of aging mice. Effect of UB on the levels of TNF-α **(H)**, IL-1β **(I)** and IL-6 **(J)** levels in the brain tissue of mice. All results are described as means ± SD (*n* = 6 per group). *^#^p* < 0.05 and *^##^p* < 0.01 vs the control group; **p* < 0.05 and ***p* < 0.01 vs the D-gal-induced aging group.

Supplementary Figure 3 UB inhibited the activation of astrocytes and microglia in D-gal-induced aging mice. Representative magnified images of immunohistochemistry of GFAP **(A)** and IBA1 **(B)** in the hippocampal CA2 region. Immunohistochemistry of GFAP **(C)** and IBA1 **(D)** in the hippocampus of each group. The statistical analysis of GFAP^+^ **(E)** and IBA1^+^ **(F)** cells per field (*n* = 6 per group). **(G)** Protein expression of GFAP and IBA1 was detected using western blotting. Quantification analysis of IBA1 **(H)** and GFAP **(I)**. β-actin served as an internal control (*n* = 6 per group). All results are described as means ± SD. *^#^p* < 0.05 and *^##^p* < 0.01 vs the control group; **p* < 0.05 and ***p* < 0.01 vs the D-gal-induced aging group.

Supplementary Figure 4 Effect of UB on memory impairment and hippocampal pathology in 2-monthand 12-month-old mice. **(A)** Escape latencies in the training trial (*n* = 12 per group). **(B)** Escape latencies of aging mice on day 5 in the training trials (*n* = 12 per group). **(C)** Time spent in the target quadrant during the probe test (*n* = 12 per group). **(D)** Number of platform location crosses by mice (*n* = 12 per group). **(E)** Representative traces on the last day of the probe test (*n* = 12 per group). **(F)** Expression of PSD95 and Syn was measured by western blotting. Quantification analysis of Syn **(G)** and PSD95 **(H)**. β-actin served as an internal control (*n* = 6 per group). **(I)** Representative Golgi staining of the hippocampal CA3 region. Representative high magnification images **(J)** and quantification of the spine number **(K)** in aging mice. All results are described as means ± SD. *^#^p* < 0.05 and *^##^p* < 0.01 vs the control group; **p* < 0.05 and ***p* < 0.01 vs the 12-month-old aging group.

Supplementary Figure 5 UB inhibited the activation of astrocytes and microglia in the hippocampus of 2-month- and 12-month-old mice. Representative immunofluorescence image of GFAP **(A)** and IBA1 **(B)** in hippocampal tissue of normal young mice and naturally senile mice. The statistical analysis of GFAP^+^ **(C)** and IBA1^+^ **(D)** cells per field (*n* = 6 per group). **(E)** Protein expression of GFAP and IBA1 was measured by western blotting. Quantification analysis of GFAP **(F)** and IBA1 **(G)**. β-actin served as an internal control (*n* = 6 per group). All results are described as means ± SD. *^#^p* < 0.05 and *^##^p* < 0.01 vs the control group; **p* < 0.05 and ***p* < 0.01 vs the 12-month-old aging group.
