# Supplementary figures and images for: The Gut Microbiota Metabolite Urolithin B Improves Cognitive Deficits by Inhibiting Cyt C-Mediated Apoptosis and Promoting the Survival of Neurons Through the PI3K Pathway in Aging Mice
Source: Front Pharmacol. 2021 Nov 15;12:768097. doi: 10.3389/fphar.2021.768097 (PMC8634731; doi:10.3389/fphar.2021.768097)

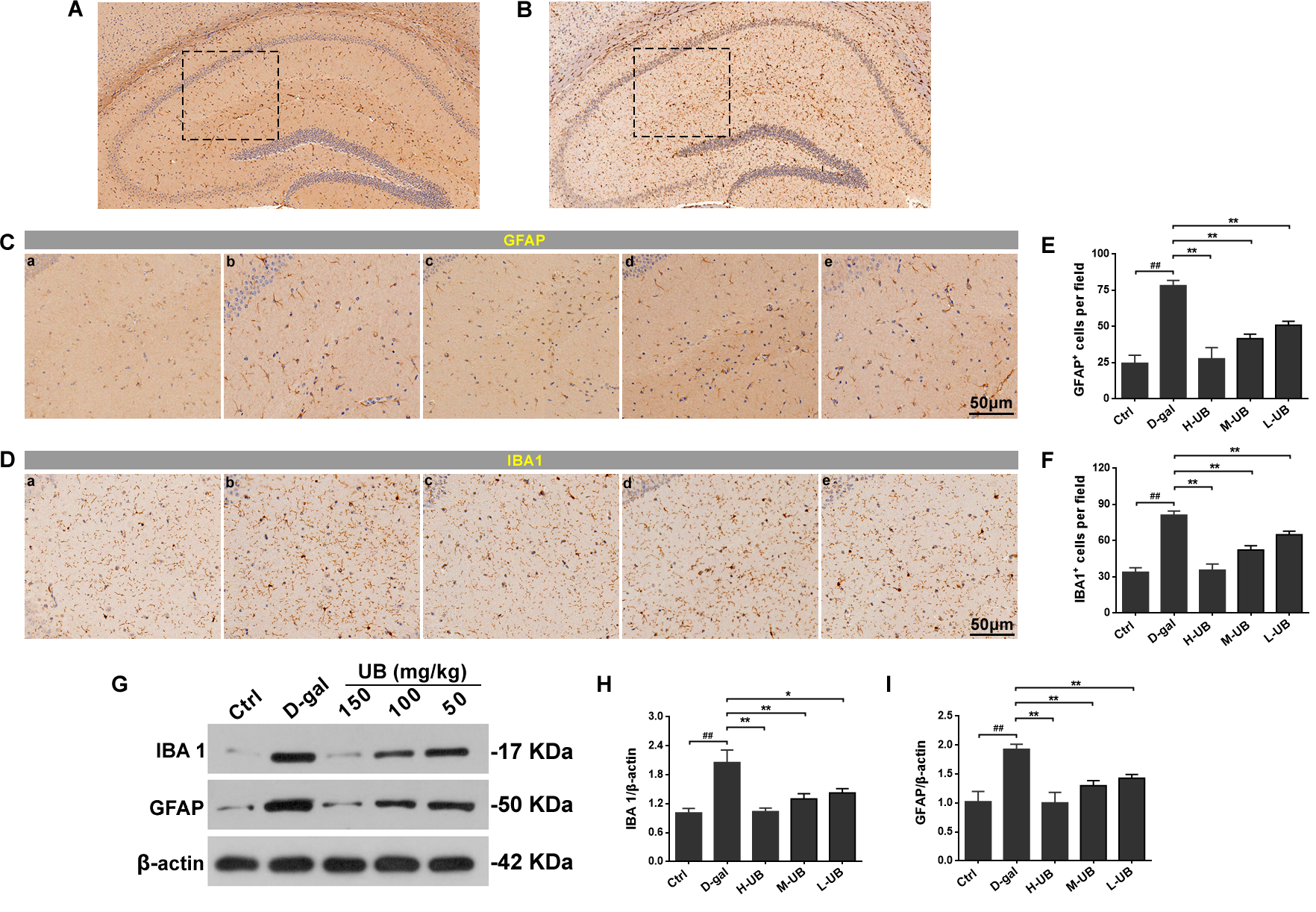

Supplement: Supplementary file 1 [file Image3.tif]

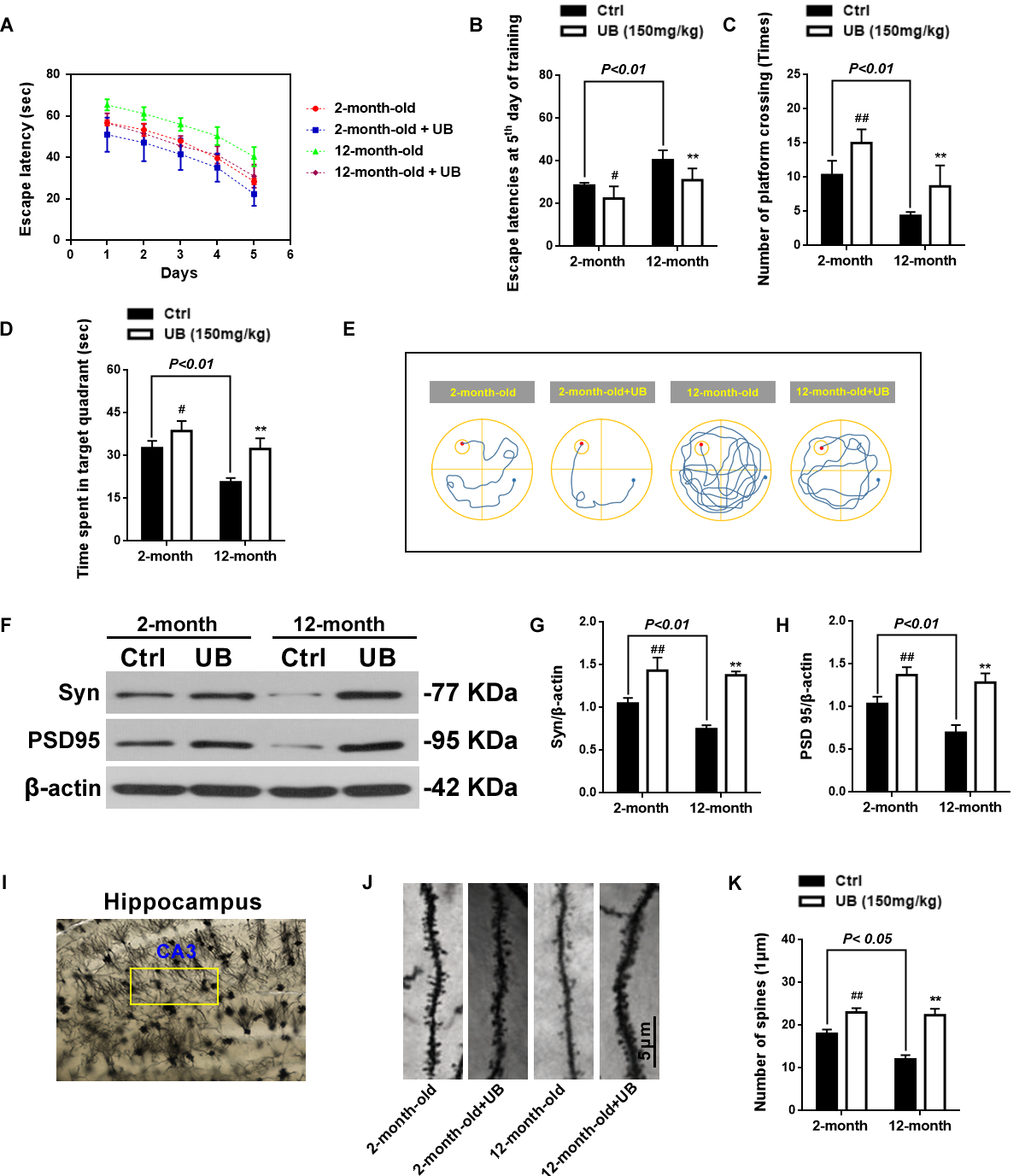

Supplement: Supplementary file 2 [file Image4.tif]

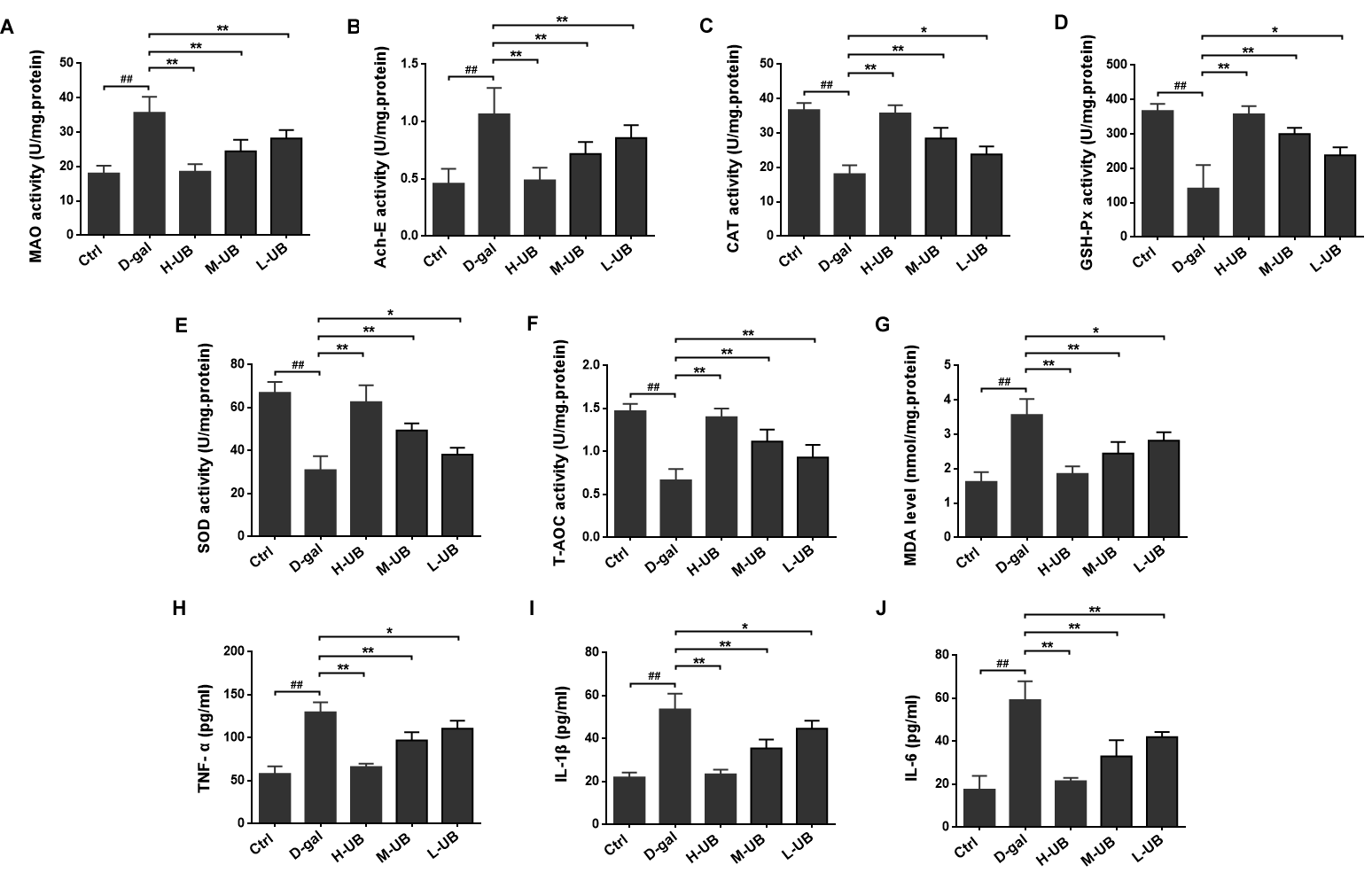

Supplement: Supplementary file 3 [file Image2.tif]

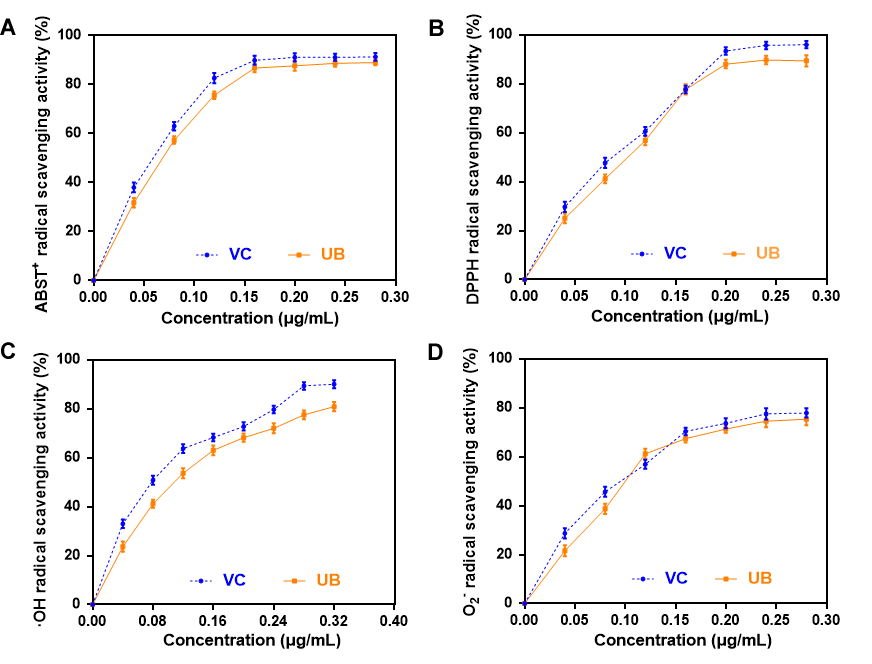

Supplement: Supplementary file 4 [file Image1.tif]

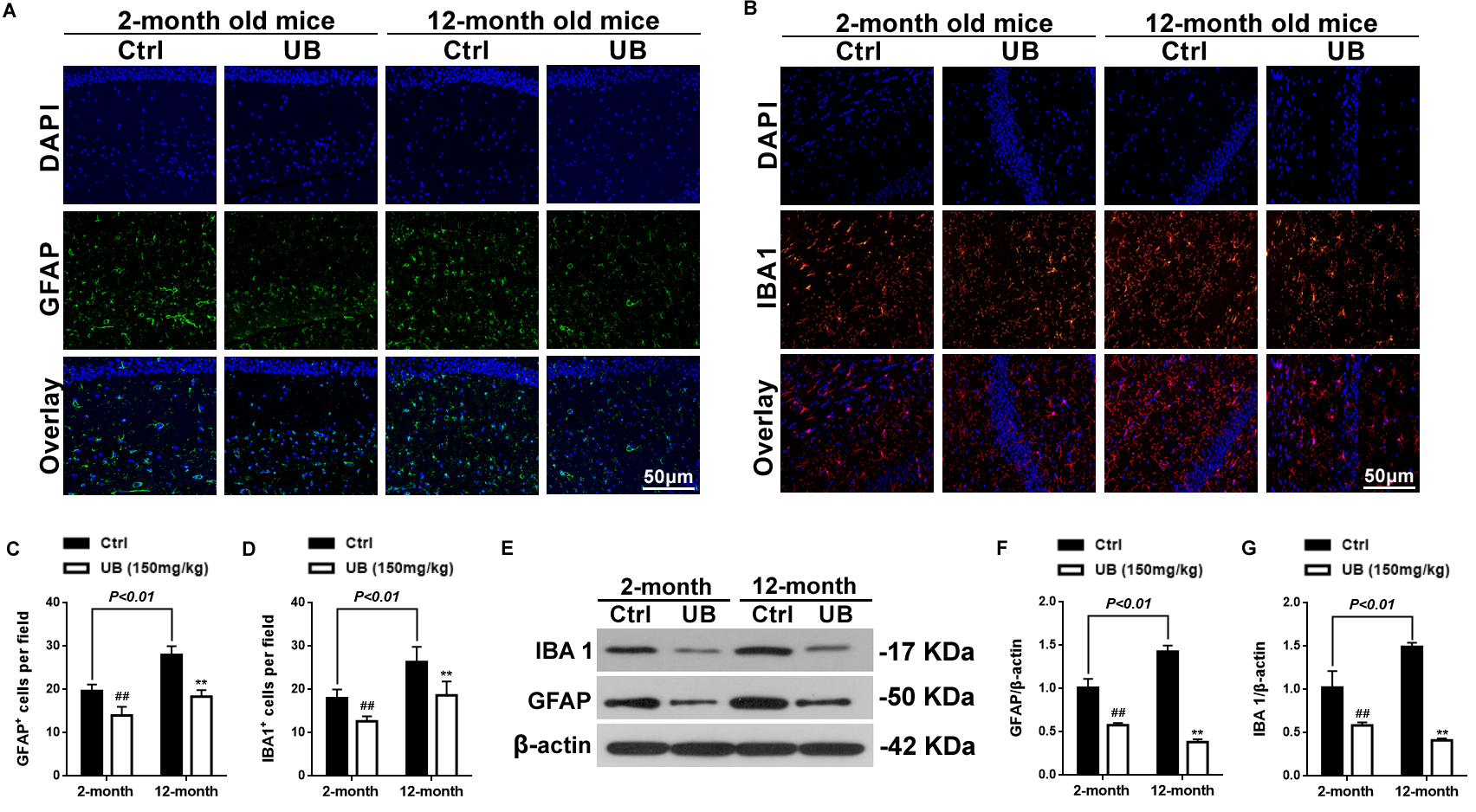

Supplement: Supplementary file 5 [file Image5.tif]
